# Nanostructures for Solar Energy Harvesting

**DOI:** 10.3390/mi14020364

**Published:** 2023-01-31

**Authors:** Mariana Sofia Santos, Ricardo A. Marques Lameirinhas, João Paulo N. Torres, João F. P. Fernandes, Catarina P. Correia V. Bernardo

**Affiliations:** 1 Department of Electrical and Computer Engineering, Instituto Superior Técnico, 1049-001 Lisbon, Portugal; 2 Instituto de Telecomunicações, 1049-001 Lisbon, Portugal; 3 Academia Militar/CINAMIL, Av. Conde Castro Guimarães, 2720-113 Amadora, Portugal; 4 IDMEC, Instituto Superior Técnico, Universidade de Lisboa, 1049-001 Lisbon, Portugal

**Keywords:** electric field concentration, nanoantennas, optoelectronic devices, photovoltaic technology, solar energy harvesting, surface plasmon polaritons

## Abstract

Renewable energy sources are becoming more and more essential to energy production as societies evolve toward a fossil-fuel-free world. Solar energy is one of the most abundant sources of green energy. Nanoantennas can be used to improve and enhance the absorption of light into a photovoltaic cell in order to generate more current. In this study, different nanoantenna structures are analysed in tandem with a silicon solar cell in an effort to improve its output. The nanoantennas studied are metallic aperture nanoantennas made up of either silver, aluminium, gold or copper. The three geometries compared are rectangular, circular and triangular. The maximum field enhancement obtained is for an aluminium rectangular nanoantenna of 50 nm thickness. Despite this, the geometry with more improvements compared with a basic silicon cell was the circle geometry with a 100 nm radius.

## 1. Introduction

The global population is estimated to reach 9.7 billion by 2050. With the growth of society comes the inherent increase in energy demand. The current production of energy relies heavily on fossil fuels, which have an undesirable environmental impact. This aligns with the energy crisis, in which the prices of fossil fuels keep rising; for example, in September 2021, the price of natural gas was as high as 6.5 times the price in 2019.

Alternatively, photovoltaic cells convert solar power into DC electric power at a somewhat low price and in a more environmentally friendly way. In addition, photovoltaic (PV) cells require very little maintenance and are robust devices, which makes them attractive in the global energy market. In particular, the global solar energy market was valued at USD 52.5 billion in 2018 and is projected to reach USD 223.3 billion by 2026 [1]. The solar radiation that reaches the earth in one day is enough to mitigate the earth’s total energy needs for a year [2].

However, in PV cells, the efficiency is limited by the bandgap of the material. Each photon with energy above the bandgap produces an electron-hole pair independently of its energy. Photons that have energy matched to the bandgap will produce the same energy delivered from high-energy photons. This limits the efficiency to approximately 30% for single-junction cells and around 55% for multi-junction cells [3].

The idea of using nanoantennas as an alternative to solar cells was first proposed by Bailey in 1972. The use of nanoantennas in photovoltaics has gained a significant amount of interest in the last few years, considering they are expected to be more efficient and inexpensive. In addition, solar nano-rectennas are not only expected to exceed PV device efficiency during the day but also possess the ability to function during the night, making them less susceptible to weather conditions [2,4].

An optical antenna, also called a nanoantenna, can enhance photo-physical phenomenons, for example, photodetection, where a photon gives rise to an electron and a hole, which is the underlying phenomenon responsible for the functioning of photovoltaic (PV) cells, thus nanoantennas can be used to enhance or improve the absorption of light into a given semiconductor [5,6,7,8,9,10].

Analogous to radio frequency (RF) antennas, nanoantennas capture the incident EM wave, either visible or infrared (IR), inducing an alternating current on the antenna surface that oscillates at the same frequency of the incident EM wave. For RF antennas, the resonance condition is achieved when the antenna size is an integer multiple of half wavelength; this is not the case for nanoantennas. At optical frequencies, metals are highly dispersive materials with finite conductivity. The displacement currents flowing along the metal have smaller wavelengths than free-space due to field penetration in the metal, appearing as plasmonic effects [5]. Additionally, the electron gas within metals can couple with light in the form of a surface wave called surface plasmon polariton (SPP) [6,7,8,11].

Considering the fact that the goal of these devices is to supply an external load, the AC current has to be rectified by a properly sized diode [9,12]. A rectenna consists of a nanoantenna, filter circuits and a rectifying diode in order to convert free-propagating electromagnetic energy into DC power [4,9].

In more recent studies, there has been an effort to combine conventional PV with nanoantennas. This has been achieved either by laying the nanoantenna on top of the PV cell or in between layers of the PV cell as an effort to increase light trapping. In this research article, several metallic nanoantennas geometries are studied in order to analyse their capability to concentrate and enhance electromagnetic radiation.

## 2. Background

### 2.1. Optical Properties of Metals

It is particularly important to characterise the metals’ optical behaviour since optical antennas rely on the resonance and plasmonic characteristics of the metals as a way to enhance radiation. The dielectric permittivity is the physical quantity that describes the metal’s behaviour under an external electric field.

The Drude–Lorentz model describes the metals’ properties at optical wavelengths by a complex dielectric function. In 1900, Paul Drude depicted the behaviour of the metals’ electrons as those of an ideal gas, hence the Drude model, also known as the free-electron model. Later, this model proved incomplete as it only describes the intraband electron transitions or the free-electron effects, conveying a metal’s behaviour at infrared wavelengths and higher, as presented in Equation (1) [8,11,13].

(1) $${\bar{\varepsilon }}_{Drude}\left(\omega \right)=1-\frac{\Omega_{p}^{2}}{\omega \left(\omega +j\Gamma_{0}\right)}$$

Subsequently, to characterise the interband electron transitions(or bound-electron effects), an equivalent to the Lorentz insulators’ model is added to the complex dielectric function, represented by Equation (2).

(2) $${\bar{\varepsilon }}_{bound}\left(\omega \right)=\sum_{n=1}^{N}\frac{f_{n}\omega_{p}^{2}}{\left(\omega_{n}^{2}-\omega^{2}\right)-j\omega \Gamma_{n}}$$

Thus, the model is expanded to the near-infrared and visible wavelength regions. The Drude–Lorentz model describes the frequency dependence of the metal’s permittivity, relating the dielectric function with the incident wave wavelength through Equation (3). In this equation, $\omega_{p}$ is the metal’s plasma frequency, and *N* is the number of resonances with resonance frequency $\omega_{n}$, with a certain oscillator’s strength $f_{n}$ and damping coefficient $\Gamma_{n}$. The $\Omega_{p}$ is the plasma frequency related to intraband transitions given by $\Omega_{p}=\sqrt{f_{0}}\omega_{p}$, with oscillator strength $f_{0}$ and damping coefficient $\Gamma_{0}$ [8,11,13].

(3) $${\bar{\varepsilon }}_{DL}\left(\omega \right)=1-\frac{\Omega_{p}^{2}}{\omega \left(\omega +j\Gamma_{0}\right)}+\sum_{n=1}^{N}\frac{f_{n}\omega_{p}^{2}}{\left(\omega_{n}^{2}-\omega^{2}\right)-j\omega \Gamma_{n}}$$

In 1998, Rakic et al. conducted a fitting of the Drude–Lorentz model parameters for several metals. However, this study focuses on four metals, silver (Ag), aluminium (Al), gold (Au) and copper (Cu), for which Table 1 contains the parameters previously mentioned [14].

### 2.2. Surface Plasmon Polaritons

A plasmon oscillation can be defined as the joint movement of conduction band electrons relative to fixed positive ions in a metal that is subjected to the incident light electric field [8,15]. When these types of oscillations are present at the interface between a conductor and a dielectric, i.e., a dielectric–metal interface, they are labelled surface plasmons [10,11].

Surface plasmons can couple the light in the form of a surface wave originating surface plasmon polaritons (SPP). The SPPs are surface electromagnetic waves that propagate along the dielectric-metal interface plane. As a result, the fields are strongly confined between the metal and the dielectric.

In order for SPPs to take place, a few conditions must be met, the metal must present electric permittivity with a negative real part at the incident light frequency. In addition, the wave vector component of the incident light that is parallel to the dielectric–metal interface must be matched with the wave number of the SPP.

For optical frequencies, few metals can satisfy the first condition, for instance, gold, silver, platinum and aluminium. Additionally, due to boundary conditions imposed by Maxwell’s equations, the SPP cannot be excited by TE waves, and it can only be excited by TM waves [8,11]. In [8], charts are presented for the four analysed metals, where the resonance properties are evaluated in function of the incident wavelength and incident angle.

In [8], a geometrical approach to SPP excitation and propagation is presented. Analysing the air–metal interfaces and the complex electrical permittivity of both media based on the SPP theory are deduced and presented in [8], and the air-metal resonances are determined.

Silver presents four resonance wavelengths, three in the UV at 49, 123, and 249 nm and one in the Visible spectra at 355 nm. On the other hand, for aluminium, there is a single resonance wavelength in the UV spectra at 119 nm. For gold, there are three resonance frequencies, two of which are in the UV, at 64 and 238 nm, and one in the visible at 536 nm. Lastly, copper presents four resonance wavelengths, two in the UV at 100 and 158 nm and two in the visible at 360 and 526 nm. These values are corroborated with the ones presented in [8].

## 3. Developed Model

### 3.1. Physics and Models

*COMSOL Multiphysics* is a finite element analysis software, a numerical method used in mathematics and engineering to determine approximate solutions for differential equations. The software *COMSOL Multiphysics* allows for several types of physics interfaces. The physics interfaces employed are the radio frequency interfaces, which compute the electric and magnetic fields in high-frequency systems. This interface possesses other subinterfaces, such as the electromagnetic waves and the frequency domain interface that solve for time-harmonic electromagnetic field distributions. A frequency domain type of study is applied for source-driven simulations for a single frequency or a sequence of frequencies.

Consequently, this physics interface solves the time-harmonic wave equation for the electric field described by Equation (4), which presents solutions in the form of Equation (5).

(4) $$\nabla \times \left(\nabla \times E\right)-k_{0}^{2}\varepsilon_{r}E=0$$

(5) $$E\left(x,y,z\right)=\bar{E}\left(x,y\right)e^{-ik_{z}z}$$

The materials’ models used are included in the COMSOL software. For metals, the 1998 Rakic fitting is used, which is found under the inorganic materials in the optical section of the materials. The employed dielectric is air, which is characterised by a constant and real permittivity equal to unity. Lastly, the semiconductor chosen is silicon (Si), which is characterised by the Aspnes and Studna fitting from 1983 [16].

**Figure 1:**
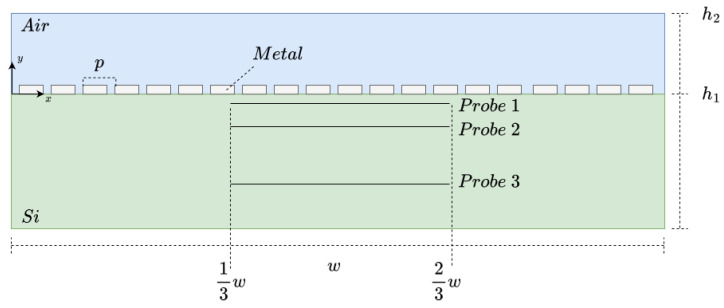
Illustrative representation of the model.

In terms of structure, the design follows the representation in Figure 1, where the width of the structure, *w*, is 9.05 μm. The design is composed of a dielectric layer (air), where an array of metal nanoantennas is contained, and a semiconductor layer. The developed model is bi-dimensional (2D) in order to reduce computation time. This approximation represents a transversal cut of a tridimensional structure, and this may be valid due to the fact that the transversal section is representative of the tridimensional device. Any two transversal planes drawn will be the same. The semiconductor layer has a height, $h_{1}$, of 2.2 μm, and the dielectric layer has a height, $h_{2}$, of $\frac{1}{3}h_{1}$. The metal nanoantenna’s height depends on the geometry in the analysis since different geometries are studied. However, the period, *p*, remains constant with a value of 450 nm. Similarly, the number of elements in the array is constant at 20 elements.

Additionally, the coordinate plane origin is defined as the dielectric–metal interface in the left corner. This implies that for *y* higher than zero, it is in the dielectric medium, and for *y* below zero, it is in the semiconductor medium.

In the frequency domain study, it is necessary to define several scattering boundary conditions. The initial values of the electric field are all set to zero. The incident electric field originates at $y=h_{2}$ and only has the *x* component different from zero, with an amplitude of 1 V/m.

#### 3.1.1. Scattering Boundary Conditions

The scattering boundary condition defines a boundary transparent for a scattered wave. The boundary is only perfectly transparent for scattered (outgoing) waves of the selected type at normal incidence to the boundary. That is, a plane wave at oblique incidence is partially reflected. At the boundary, the wave is decomposed into two components, perpendicular and parallel. The perpendicular component is absorbed at the boundary. Meanwhile, the parallel component keeps propagating. This condition is applied for x=0, x=w, and $y=-h_{1}$, giving rise to well-defined boundaries.

#### 3.1.2. Probe Placement

A crucial step in the model design is the definition of the three probes present in Figure 1. The distance from the dielectric–semiconductor interface is defined following the radiation zones for near-field and far-field, where each probe corresponds to the limit of the zones. Hence, the minimum wavelength considered for the delimitation of the radiation zones was 200 nm, even though some of the studies include shorter wavelengths that would yield distances too close to the interface for the overall range of frequencies studied. Moreover, the maximum wavelength studied is 700 nm, the upper limit of the visible spectrum. Consequently, probe 1 is defined at $d_{1}=-\frac{200}{2\pi }$ nm, probe 2 is located at $d_{2}=-200$ nm, and lastly, probe 3 is at $d_{3}=-1500$ nm since it is twice as large as 700 nm. As for the length of the probes, they are all $\frac{1}{3}w$ long, spanning from 3 to 6 μm, in order to avoid the waves that might be reflected from the boundaries previously defined. That is, if the probe spanned throughout the entire semiconductor layer, from boundary to boundary, the electric field maxima would have probably been in the boundary points due to the interaction between the reflected waves at the boundary.

#### 3.1.3. Frequency Ranges

As formerly mentioned, the frequency domain study is applied to the simulation for a sequence of frequencies. The frequency ranges were all obtained similarly.

Furthermore, the visible spectrum ranges from 400 to 700 nm, noting with special importance that the Sun’s radiation peak intensity is at a wavelength of approximately 500 nm. Consequently, a linearly spaced vector starting from 200 to 700 nm, with a spacing of 50 nm, is created as a start. The previously determined air–metal resonances are then added to this vector, depending on which metal is present in the structure. The resulting vector is then converted to THz, and the simulation frequency ranges are defined.

#### 3.1.4. Nanoantenna Geometry

**Figure 2:**
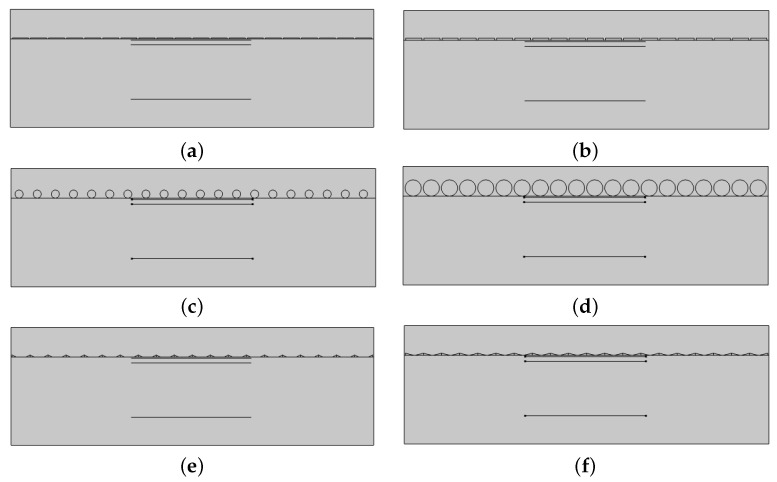
The different structure designs. (**a**) Rectangular structure with 20 nm thickness. (**b**) Rectangular structure with 50 nm thickness. (**c**) Circular structure with 100 nm radius. (**d**) Circular structure with 200 nm radius. (**e**) Triangular structure with 100 nm base and 50 nm height. (**f**) Triangular structure with 200 nm base and 50 nm height.

The study focuses on three aperture metal nanoantenna geometries rectangular, circular and triangular. In the rectangular geometry, the simulations are conducted for a metal thickness of 20 nm, present in Figure 2a, and 50 nm, as seen in Figure 2b, with a width of 400 nm. On the other hand, in the circular geometry, the circle is tangent to the dielectric–semiconductor interface, and it was tested for a radius of 100 nm (Figure 2c) and 200 nm (Figure 2d). Lastly, the triangular geometry, where the array consists of two triangles with base *b* side by side, with a base of 100 and 200 nm, is illustrated in Figure 2e,f, respectively.

#### 3.1.5. Mesh

The mesh defines the discretisation of the geometry, which divides the geometry into small units of simple shapes (mesh elements), allowing the computation of approximate solutions over each mesh element rather than the entire geometry. The type of mesh element is triangular, with a maximum element size of 6.767 × $10^{-7}$ m, a minimum element size of 2.03 × $10^{-8}$ m, and a maximum element growth rate of 1.5.

## 4. Results

### 4.1. Si Cell without Nanoantennas

**Figure 3:**
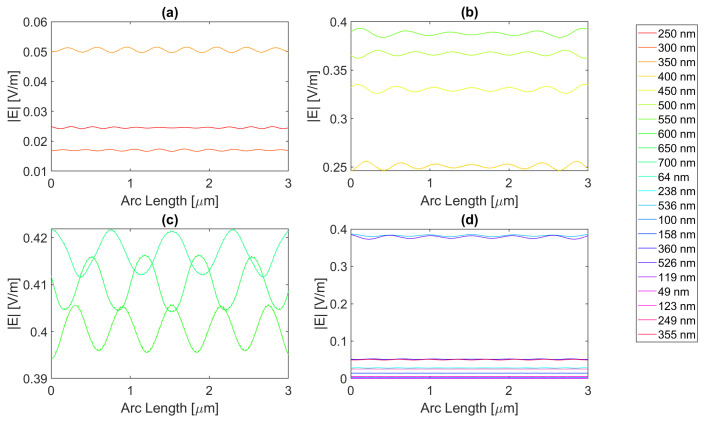
Electric field at probe 1 for the stand-alone Si cell.

To set a comparative base, the case without any nanoantenna is simulated for the first probe, and the results are shown in Figure 3. The analysed values are the electric field and the integral of the field along the probe (line integral). For solar technology, it is essential to study these values because a larger field translates into a higher concentration, which in turn can lead to the possibility of reducing the area of the cell to produce the same amount of energy.

Analysing the maximum electric field value for the standalone Si cell, at a wavelength of 700 nm results as $|E_{max}|\;=\;4.22\times \;10^{-1}$ V/m and $\int E\;dx\;=\;1.250\times \;10^{-6}$.

### 4.2. Rectangular

Four rectangular studies have been carried out for the 20 nm thickness design, shown in Figure 2a, and the 50 nm thickness design, shown in Figure 2b, for silver, aluminium, gold and copper.

**Figure 4:**
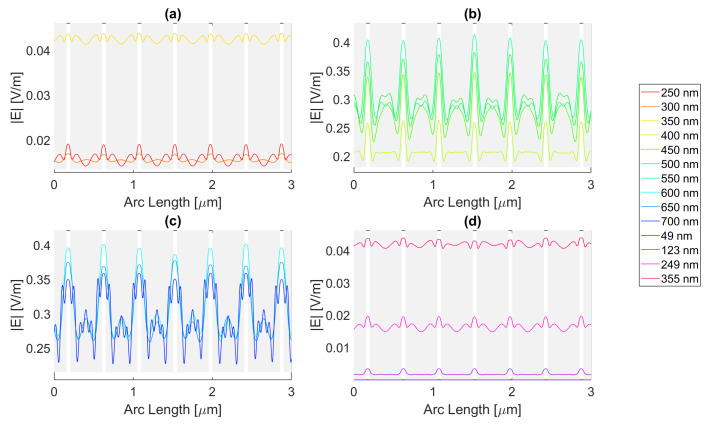
Electric field for silver 20-nm-thick rectangular nanostructure—Probe 1.

**Figure 5:**
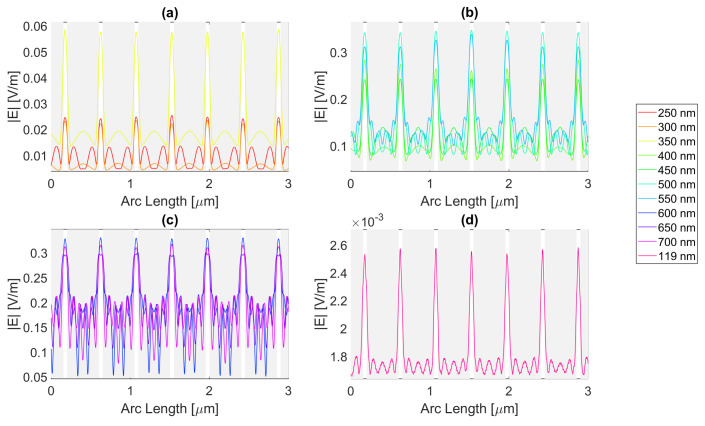
Electric field for aluminium 20-nm-thick rectangular nanostructure—Probe 1.

**Figure 6:**
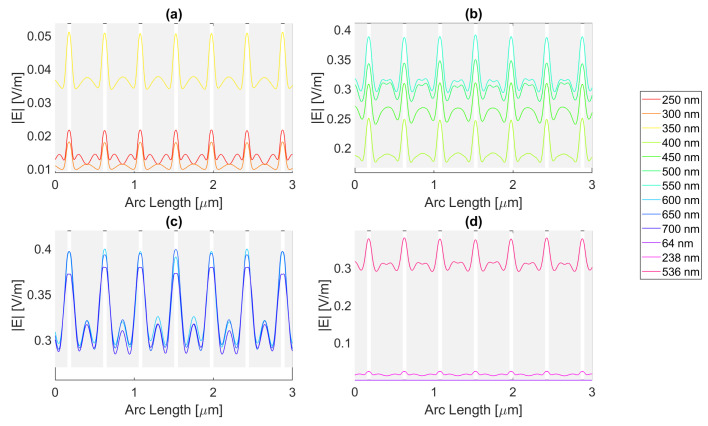
Electric field for gold 20-nm-thick rectangular nanostructure—Probe 1.

**Figure 7:**
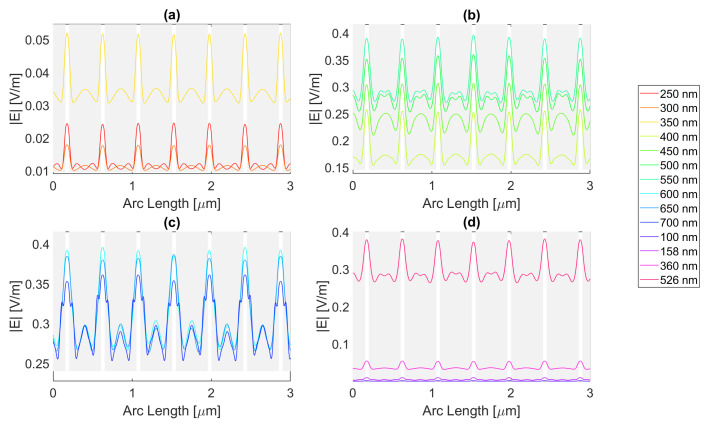
Electric field for copper 20-nm-thick rectangular nanostructure—Probe 1.

For a rectangular nanoantenna with a 20 nm thickness, it is possible to observe that there is field confinement and concentration in the slits since the incident electric field on the sides of the nanoantenna is reflected into the slit area, thus causing field concentration and a confinement effect in this area. Therefore, in probe 1, most curves are a perfect replica of the slit, i.e., the field peaks on the slit, and decrease considerably at the end of the slit. However, at certain wavelengths, the metallic antenna does not fully reflect the electric field and may even transmit it. This phenomenon occurs near the resonance wavelengths, giving rise to electric field peaks in probe 1, where one would expect to have continuously decreasing values, as seen in Figure 4, Figure 5, Figure 6 and Figure 7.

For silver, represented in Figure 4, the largest peaks are reached for wavelengths between 500 and 650 nm, and the largest peak is reached for a wavelength of 550 nm with a field value of 4.13 × $10^{-1}$ V/m. It is, therefore, still lower than the field obtained for silicon. Additionally, it should be noted that, for an incident field of 700 nm, the electric field presents peaks near the slit boundaries, a pattern that is unique for the frequencies studied, present in Figure 4c, repeating the pattern in the nanoantenna with smaller values. As for the field integral, at a wavelength of 600 nm it reaches its maximum equal to 9.29 × $10^{-7}$. The results for all frequencies studied can be found in the Appendix A, Table A1 for probe 1, Table A2 for the second probe, and Table A3 for probe 3. Comparing the values relative to probe 1, probe 2, and probe 3, the electric field and field integral have all progressively decreased as the distance from the antennas grows, most likely meaning the electric field has been absorbed by the semiconductor.

Aluminium, on the other hand, present in Figure 5, has its only resonance frequency in the UV spectrum, at 119 nm, and although at this wavelength, the absorbed field is small, it is still possible to observe transmission through the antenna. Interestingly, for the larger wavelengths, the electric field norm pattern maintains the maximum values practically constant at the slit, in contrast with the antenna area, which experiences a greater number of peaks, where the maximums are greater, and the minimums are lower, i.e., the electric field norm showcases a greater oscillation in values. For this structure, with a thickness of 20 nm, the maximum electric field norm value is 3.47 × $10^{-1}$ V/m for a 500 nm incident wavelength, and the maximum integral is 5.97 × $10^{-7}$ for a 650 nm wavelength. The results for all frequencies studied can be found in Appendix A Table A4, Table A5 and Table A6 for probes 1, 2, and 3, respectively. From the values in these tables, it is possible to conclude that for a rectangular geometry with 20 nm thickness for the aluminium antenna, the electric field and its integral decrease from the first probe to the second and from the second to the third, conveying the absorption of the electric field by silicon.

On the other hand, the gold antenna reaches a maximum electric field of 4.00 × $10^{-1}$ V/m and field integral of 9.87 × $10^{-7}$ at a 600 nm wavelength, being the design of 20 nm thickness that has a maximum at the largest wavelength, as represented in Figure 6c. In Figure 6b, for wavelengths near the resonance wavelength 536 nm, from 450 to 700 nm, the field on the antenna area is transmitted due to extraordinary optical transmission (EOT). The results for all frequencies studied can be found in Table A7 for probe 1, Table A8 for probe 2, and Table A9 for probe 3. As the distance from the slit grows, the electric field and its integral decrease for this gold nanoantenna, implying the absorption by the semiconductor of the electric field.

Lastly, in Figure 7, for the wavelengths between 450 and 700 nm, it can be seen that transmission through the metal occurred, and this increased as the wavelength approached the resonance at 526 nm, reaching a maximum field norm of 3.97 × $10^{-1}$ at 550 nm and a maximum field integral of 9.33 × $10^{-7}$ at 600 nm. It is, therefore, still lower than the field obtained for silicon.

The results for all frequencies studied can be found in Table A10, Table A11, and Table A12 for probe 1, 2, and 3, respectively. Comparing the values relative to probe 1, probe 2, and probe 3, the electric field and field integral all progressively decreased as the distance from the antennas increased, most likely meaning the electric field was absorbed by the semiconductor.

Compared with the originally considered silicon cell, for a rectangular nanoantenna with a thickness of 20 nm, there were no improvements in terms of electric field enhancement.

**Figure 8:**
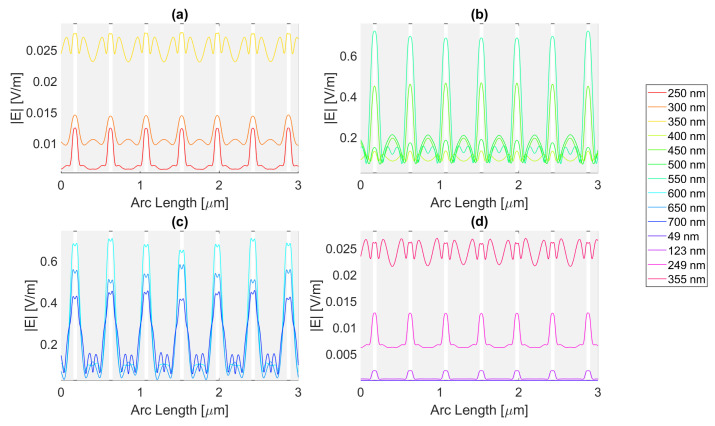
Electric field for silver 50-nm-thick rectangular nanostructure—Probe 1.

**Figure 9:**
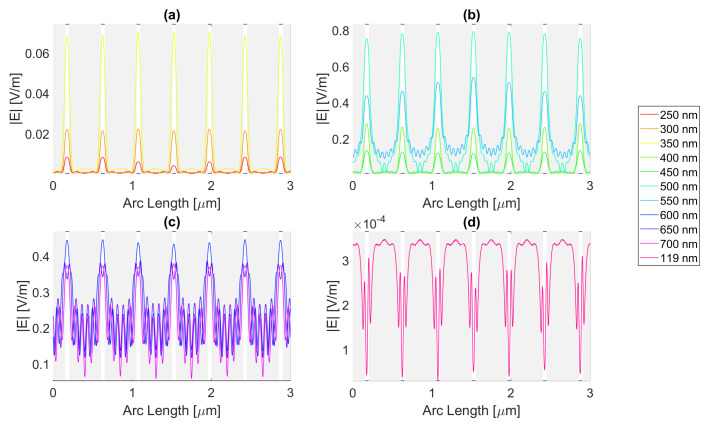
Electric field for aluminium 50-nm-thick rectangular nanostructure—Probe 1.

**Figure 10:**
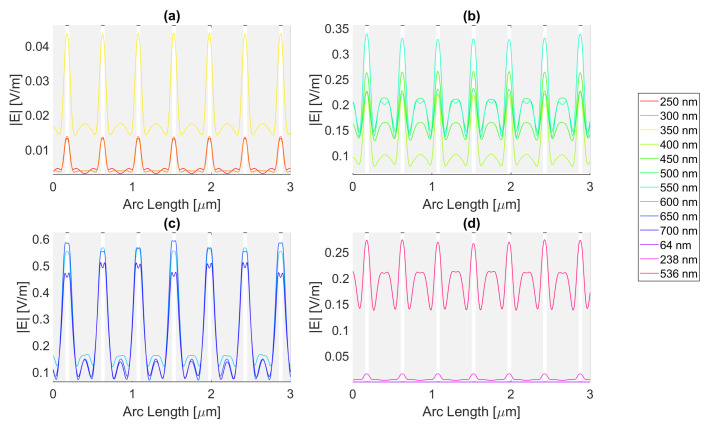
Electric field for gold 50-nm-thick rectangular nanostructure—Probe 1.

**Figure 11:**
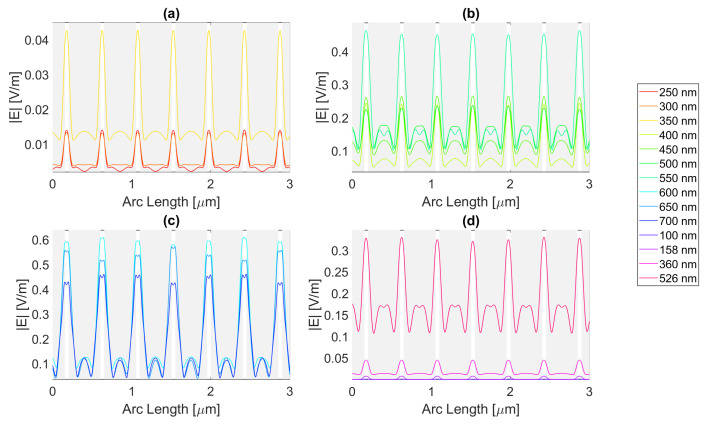
Electric field for copper 50-nm-thick rectangular nanostructure—Probe 1.

Thereafter, for a nanoantenna of 50 nm thickness, the same metals were simulated, yielding Figure 8, Figure 9, Figure 10 and Figure 11. In this case, for Ag and Al, the electric field maximum maintained the same wavelength, in contrast to Au and Cu. The generic shape of the radiation pattern remains somewhat similar. The biggest difference is that the new maximum values are all greater than the 20 nm values and superior to the original silicon cell field obtained.

In such circumstances, Ag reaches a maximum electric field, $|E_{max}|$, of 7.22 × $10^{-1}$ V/m for a wavelength, λ, of 550 nm, greater than the stand-alone silicon cell electric field maximum value, $|E_{max}{|}^{Si}$. However, the integral of the electric field along the probe, ∫Edx, 8.16 × $10^{-7}$ is smaller than that of the stand-alone Silicon cell, ${\int E\;dx}^{Si}$.

Figure 8 shows that in (a) in the UV region, the radiation pattern follows the typical pattern for the shadow zone, except at 350 nm, which again is near the resonance wavelength, and it has an analogous shape to the resonance wavelength, 355 nm, in (d). Whereas, in (c), in the slit, there are two peaks in the slit region, and for 700 nm, there are also two peaks in the antenna region. The results for all wavelengths studied can be found in Table A1, Table A2, and Table A3, for probes 1, 2, and 3, respectively. The electric field intensity norm decreases from probe 1 to 2 and from probe 2 to 3. On the other hand, the field integral for wavelengths from 550 to 650 nm increases from probe 1 to 2, which means that the electric field from additional optical paths can propagate into the plane of the probe, not indicating a necessary increase in the peak electric field obtained for that frequency. For the aluminium antenna, there is a greater field concentration evident in the slit area at 500 nm, in Figure 9b, where the $|E_{max}|$ is 7.95 × $10^{-1}$ V/m. However, similarly to silver, the ∫Edx is lower than ${\int E\;dx}^{Si}$, it is 7.74 × $10^{-7}$ at 600 nm. Unlike the 20 nm structure, the peaks in the antenna region start appearing earlier, not as pronounced, at around 500 nm, remaining below 2 × $10^{-1}$ V/m. In Figure 9c, the peaks are more pronounced, with an approximate value of 3 × $10^{-1}$ V/m at 650 nm. The results for all wavelengths studied can be found in Table A4, Table A5, and Table A6, for probes 1, 2, and 3, respectively. From probe 1 to 2, and from probe to 2 to 3, both the electric field and integral decrease as the silicon absorbs the electric field.

It is noticeable in Figure 10c that the double peaks in the slit seem to also appear for the gold nanoantenna, at 650 and 700 nm, similar to silver, leading to an $|E_{max}|$ of 5.94 × $10^{-1}$ V/m at 650 nm, and a maximum ∫Edx of 8.02 × $10^{-7}$ at 600 nm. Even though gold has a resonance at 536 nm and around that wavelength, it is evident that the transmission through the metal is more significant, and it is still insufficient to produce a greater electric field or electric field integral in the slit. The results for all wavelengths studied can be found in Table A7, Table A8, and Table A9, for probes 1, 2, and 3, respectively.

In comparison, probe 2 has smaller values of the electric field and integral than probe 1, except for the wavelengths of 650 and 700 nm, for which the field integral increases, meaning that for the equivalent frequencies, the electric field propagates inside the semiconductor through additional optical paths into the probes’ plane and does not result in a necessary increase in the maximum electric field. From probe 2 to 3, both the electric field intensity and integral decrease as the silicon absorbs the electric field.

Finally, for copper, the $|E_{max}|$ is 6.09 × $10^{-1}$ V/m, and the maximum ∫Edx is 7.65 × $10^{-7}$ at 600 nm.

As with silver and gold, in Figure 11, copper shows two peaks for wavelengths between 600 and 700 nm at the slit. Since the design is thicker, there is more antenna side area, which results in more reflected radiation.

Moreso, for wavelengths near the resonance at 526 nm, the maximum electric field remains relatively unchanged and is even lower in some cases due to the thicker sheet of metal decreasing the transmission through it. The results for all wavelengths studied can be found in Table A10, Table A11, and Table A12, for probes 1, 2, and 3, respectively. From probe 1 to 2, both the electric field and integral decrease, except at wavelengths of 600 and 650 nm, where the field integral increases. In contrast, from probe 2 to 3 for all wavelengths, the electric field intensity and integral both decrease.

In an effort to increase transmission through the metal structures, two metals were combined.

### 4.3. Circular Nanostructure

The circular design is introduced in order to try to improve both the electric field concentration and also the value of the field integral. The types of simulated structures are presented in Figure 2c,d. The slit consists of the outline of two consecutive circles, resembling a triangular shape with rounded sides. This arrangement may lead to more field reflection from the side of the antennas since the circles are only tangent to the semiconductor, in contrast to the rectangular antennas, which were entirely in contact with the top of the semiconductor.

**Figure 12:**
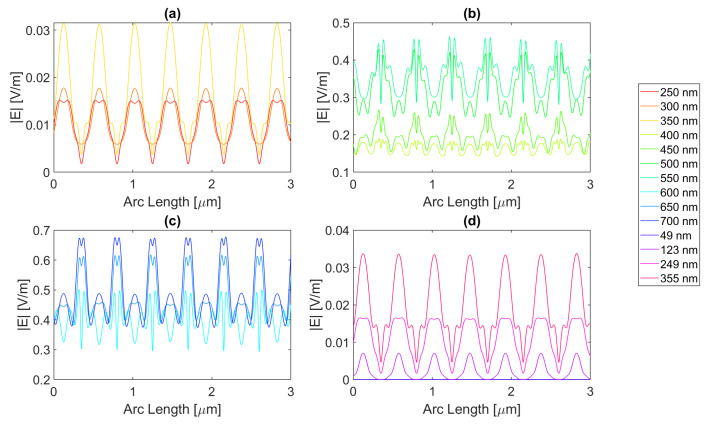
Electric field for the silver circular nanostructure with a 100 nm radius—Probe 1.

Starting by the antenna with a 100 nm radius, it is evident that for certain frequencies, the electric field present in the silicon perfectly replicates the aperture described above, just as in Figure 12 for a wavelength of 123 nm; although there is a small electric field at this wavelength, it perfectly illustrates the shadow zone because the triangular shape with rounded sides is evident. The other frequencies generally have a similar pattern. In addition, in the visible zone, the slits interfere with each other’s field, sometimes creating “shadows” where the field has local minimums, whereas at other wavelengths, it has maximums, for example, the 600 vs. 300 nm curve. In fact, for the visible wavelengths in Figure 12b,c, the electric field maximum peaks are achieved under the metal zone, while for UV, it is in the slit region, i.e., (c) and (d).

For the circular antenna with a 100 nm radius, the maximum electric field achieved for silver is 6.77 × $10^{-1}$ V/m, with an integral of the field 1.48 × $10^{-6}$ at 700 nm. Both of these are greater than the values for the silicon cell without a nanostructure. The results for all frequencies studied can be found in the tables in Appendix A. From probe 1 to probe 2, there is an increase in the electric field intensity for 600 and 650 nm wavelengths and a decrease in its integral. From probe 2 to probe 3, there is an increase in the electric field intensity at 700 nm wavelength reaching a value as high as 6.82 × $10^{-1}$ V/m, even higher than in probe 1, while it decreases for the remaining wavelengths. This might indicate that this geometry and material are capable of inducing a long-distance field concentration on the silicon.

**Figure 13:**
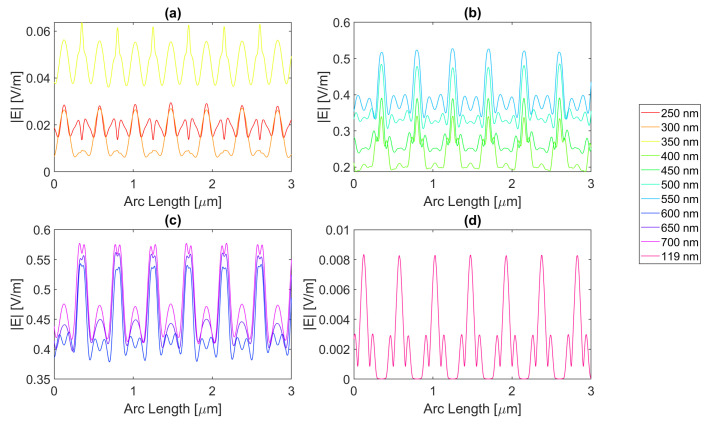
Electric field for the aluminium circular nanostructure with 100 nm radius—Probe 1.

**Figure 14:**
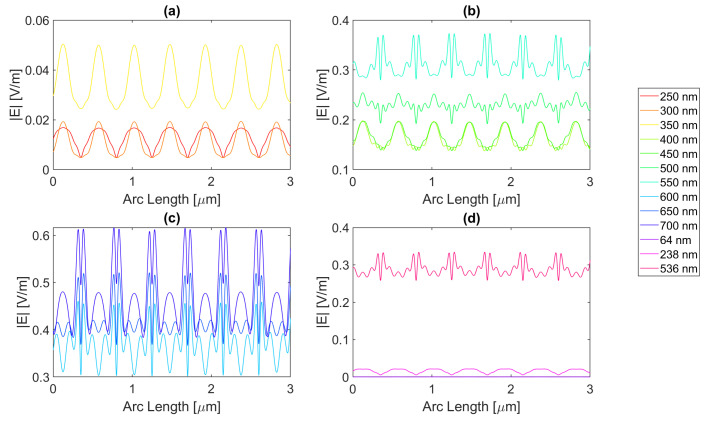
Electric field for the gold circular nanostructure with a 100 nm radius—Probe 1.

Figure 13 represents the electric field intensity along probe 1 for aluminium. The highest electric field attained is 5.78 × $10^{-1}$ V/m and an integral of 1.43 × $10^{-6}$ at 700 nm, representing an improvement from the basic cell. The results of the studied wavelengths can be found in Table A4, Table A5, and Table A6 for probes 1, 2, and 3, respectively. From probes 1 to 2, for wavelengths between 600 and 700 nm, the electric field intensity increases, and it keeps increasing for 700 nm in probe 3, reaching 6.18 × $10^{-1}$ V/m in the third probe. As for the electric field integral, it decreases from probe to probe. The electric field intensity for gold is illustrated in Figure 14, showing that the best electric field achieved is 6.17 × $10^{-1}$ V/m at 700 nm with a field integral of 1.42 × $10^{-6}$, once again showing improvement in terms of both quantities. For the studied wavelengths, the results studied can be found in Table A7 for probe 1, Table A8 for probe 2, and Table A9 for probe 3. From probes 1 to 2, the electric field intensity increases for wavelengths between 500 and 650 nm, while the field integral decreases. In contrast, from probes 2 to 3, both quantities decrease except for a wavelength of 700 nm in which the intensity increases reaching 6.45 × $10^{-1}$ V/m.

**Figure 15:**
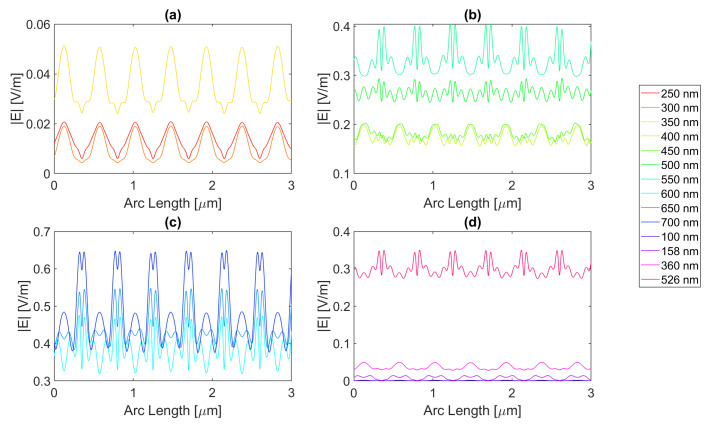
Electric field for the copper circular nanostructure with a 100 nm radius—Probe 1.

Lastly, copper yields an electric field curve illustrated in Figure 15, achieving an intensity as high as 6.49 × $10^{-1}$ V/m and an integral of 1.45 × $10^{-6}$ at 700 nm, making it the second highest intensity reached with the circular structure with a 100 nm radius. For the studied wavelengths, the results studied can be found in Table A10 for probe 1, Table A11 for probe 2, and Table A12 for probe 3. The electric field intensity increases for wavelengths ranging from 500 to 650 nm, from probe 1 to 2, while the integral decreases. At 700 nm from probe 2 to 3, the intensity increases, yet for the remaining wavelengths, it decreases, and so does the integral.

**Figure 16:**
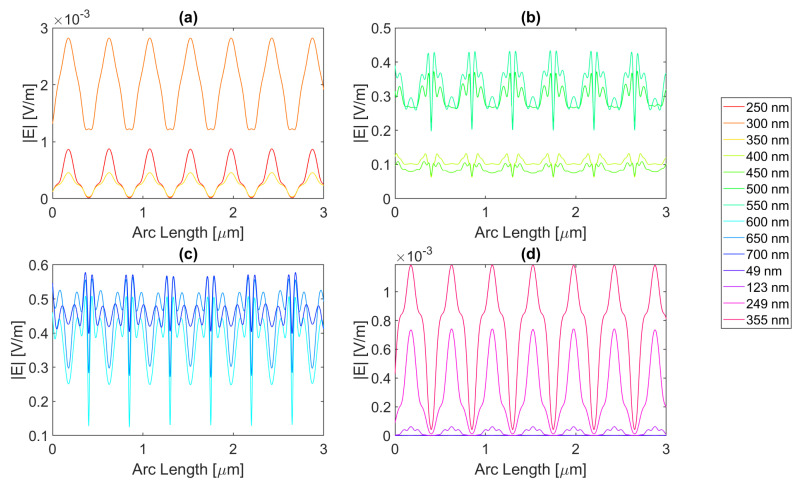
Electric field for the silver circular nanostructure with a 200 nm radius—Probe 1.

**Figure 17:**
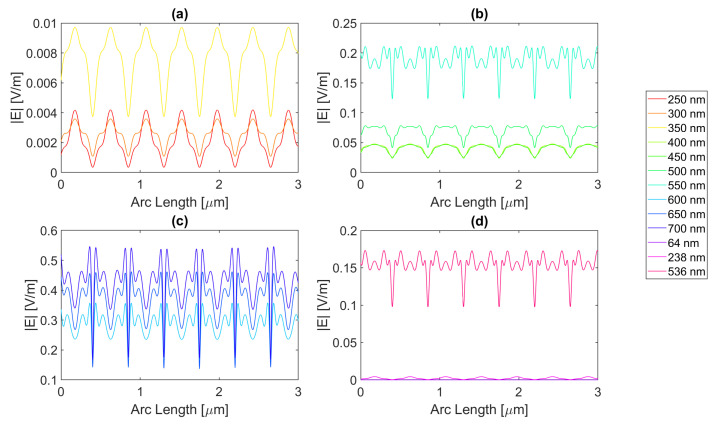
Electric field for the gold circular nanostructure with 200 nm radius—Probe 1.

**Figure 18:**
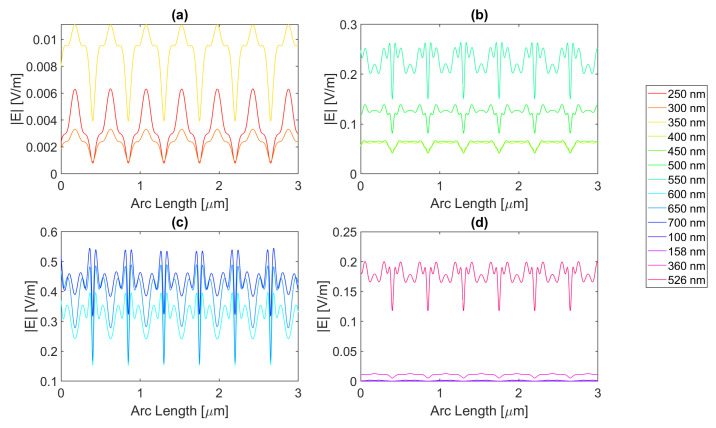
Electric field for the copper circular nanostructure with a 200 nm radius—Probe 1.

**Figure 19:**
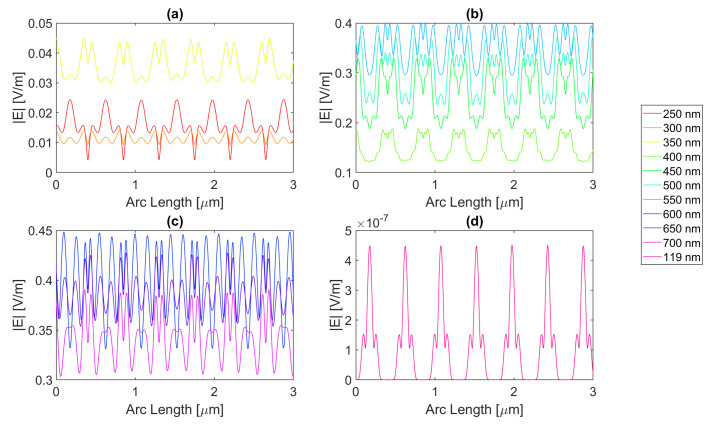
Electric field for the aluminium circular nanostructure with a 200 nm radius—Probe 1.

Now, moving on to the circular nanoantenna with a 200 nm radius, the electric field intensity characteristics at probe 1 is in Figure 16, Figure 17, Figure 18 and Figure 19. Even though the radius increased, the displacement between the centre of the circles is maintained, which leads to an overall smaller aperture. For this structure, all of the metals showed improvements in electric field intensity over the simple silicon cell. For silver, the maximum electric field strength achieved is about 5.78 × $10^{-1}$ V/m for a wavelength of 700 nm, with a field integral of 1.40 × $10^{-6}$, the highest value achieved for this geometry. The results of the studied wavelengths can be found in Table A1, Table A2, and Table A3 for probes 1, 2, and 3, respectively. The electric field intensity increases from probe 1 to probe 2 for particular wavelengths, such as 500, 600, and 700 nm, despite the field integral decreasing for all wavelengths. As for probe 3, both the electric field intensity and integral have decreased, alluding to the absorption of the electric field by the semiconductor material.

Now for gold, the highest field attained is approximately 5.46 × $10^{-1}$ V/m, with a 1.29 × $10^{-6}$ integral, for a wavelength of 700 nm. The results of the studied wavelengths can be found in Table A7, Table A8, and Table A9 for probes 1, 2, and 3, respectively. For the range of wavelengths between 500 and 650 nm, the electric field intensity increases from probe 1 to probe 2, yet the field integral always decreases. At 700 nm, the intensity also increases for the third probe, while the integral decreases for all wavelengths.

For copper, the electric field peak is similar to gold, and even at the same wavelength, it is 5.45 × $10^{-1}$ V/m, with a 1.33 × $10^{-6}$ integral. The results of the studied wavelengths can be found in Table A10, Table A11, and Table A12 for probes 1, 2, and 3, respectively. For the range of wavelengths between 500 and 700 nm, the electric field intensity increases from probe 1 to probe 2; however, the field integral always decreases. In the third probe at 700 nm, the intensity also increases, while the integral decreases for all wavelengths.

Even though aluminium does show an improvement in the maximum electric field, being 4.49 × $10^{-1}$ V/m, the integral does not, as it falls just under the basic Si cell with a value of 1.20 × $10^{-6}$, for a 600 nm wavelength. The results of the studied wavelengths can be found in Table A4, Table A5, and Table A6 for probes 1, 2, and 3, respectively. For the range of wavelengths between 550 and 700 nm, the electric field intensity increases from probe 1 to probe 2, contrary to the field integral, which always decreases. At 700 nm, the intensity also increases for the third probe, while the integral decreases for all wavelengths.

### 4.4. Triangular Nanostructure

In this section, the triangular design is introduced, the simulated structures are present in Figure 2e,f.

**Figure 20:**
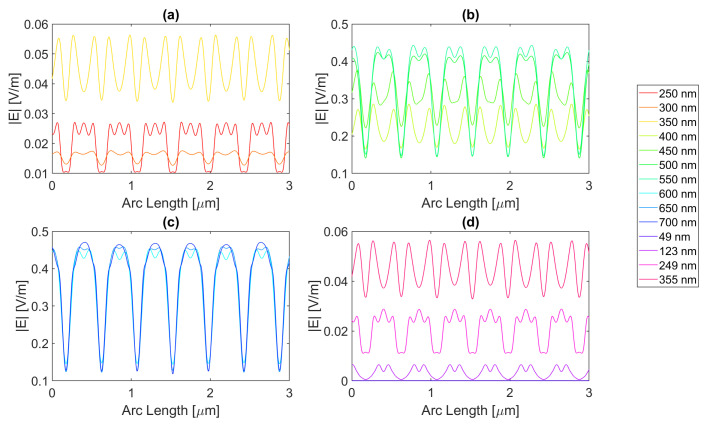
Electric field for the silver triangular nanostructure with a 100 nm base—Probe 1.

Starting with the triangular structure with a base of 100 nm, all the distinct metal structures offer an enhancement of electric field values, but none in terms of the field integral. This might be due to the aperture’s trapezoidal shape, where the smaller base of 250 nm is the top of the semiconductor, and its legs are the contour of the triangular antennas, resulting in the reflection of the electric field into the air instead of the semiconductor. For the 200 nm base structure, the smaller base of the trapezoid is instead 50 nm wide, that is, the slit. In the silver triangular antenna with a base of 100 nm, with an electric field intensity pattern, as seen in Figure 20, the peak electric field intensity is 4.71 × $10^{-1}$ V/m with an integral of 1.09 × $10^{-6}$ at 700 nm. The results of the studied wavelengths can be found in Table A1, Table A2, and Table A3 for probes 1, 2, and 3, respectively. The electric field intensity increases from probe 1 to probe 2 for a range of wavelengths, from 550 to 700 nm, despite the field integral decreasing for all wavelengths. As for probe 3, both the electric field intensity and integral have decreased, indicating the absorption of the electric field by the semiconductor material.

**Figure 21:**
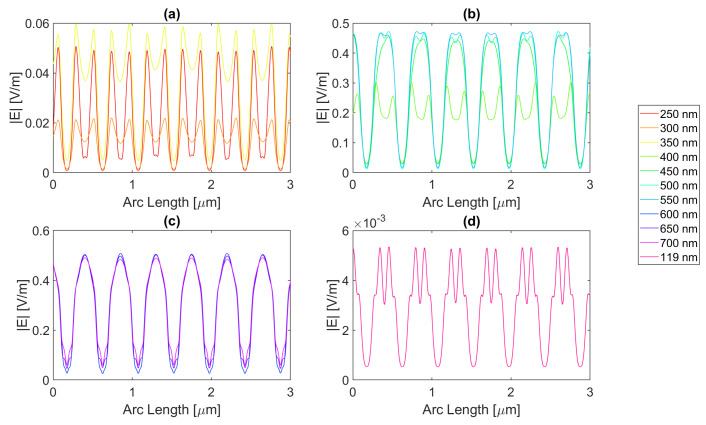
Electric field for the aluminium triangular nanostructure with a 100 nm base—Probe 1.

As for aluminium, illustrated in Figure 21, the achieved field is higher, but the integral is lower, with values of 5.08 × $10^{-1}$ V/m at 650 nm and 9.14 × $10^{-7}$ at 700 nm, respectively. The results of the studied wavelengths can be found in Table A4, Table A5, and Table A6 for probes 1, 2, and 3, respectively. The electric field intensity increases from probe 1 to probe 2 for a range of wavelengths, from 500 to 700 nm, despite the field integral decreasing for all wavelengths. As for probe 3, both the electric field intensity and integral have decreased, indicating the absorption of the electric field by the semiconductor material.

**Figure 22:**
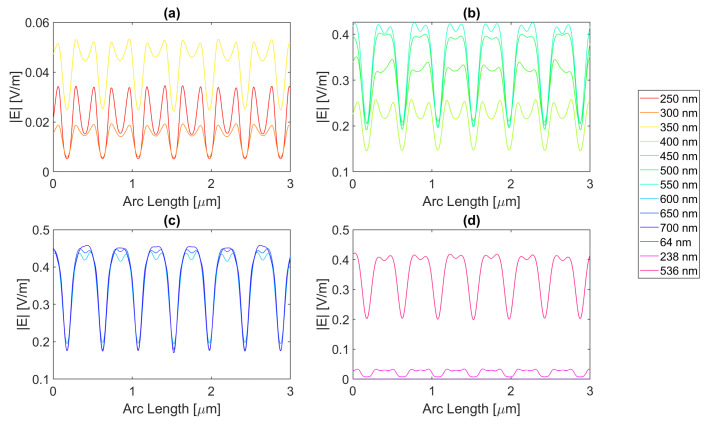
Electric field for the gold triangular nanostructure with a 100 nm base—Probe 1.

**Figure 23:**
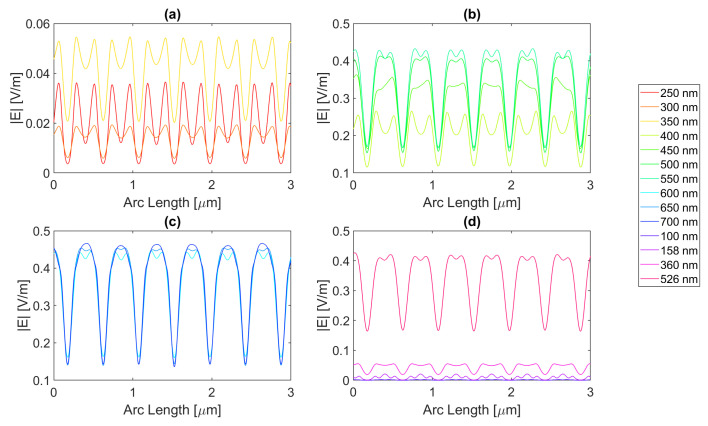
Electric field for the copper triangular nanostructure with a 100 nm base—Probe 1.

Gold and copper, with electric field intensity curves as seen in Figure 22 and Figure 23, present similar maximum values with 4.58 × $10^{-1}$ and 4.66 × $10^{-1}$ V/m electric field intensities and, 1.11 × $10^{-6}$ and 1.09 × $10^{-6}$ field integrals, respectively, at a 700 nm wavelength. The results of the studied wavelengths for gold can be found in Table A7, Table A8, and Table A9 for probes 1, 2, and 3, respectively.

The electric field intensity increases from probe 1 to probe 2 for a range of wavelengths, from 550 to 700 nm, despite the field integral decreasing for all wavelengths. As for probe 3, both the electric field intensity and integral have decreased, implying the absorption of the electric field by the semiconductor material. While for copper, the results for the studied wavelengths are in Table A10, Table A11, and Table A12 for probes 1, 2, and 3, respectively. The electric field intensity increases from probe 1 to probe 2 for a range of wavelengths, from 550 to 700 nm, yet the field integral decreases for all wavelengths. As for probe 3, both the electric field intensity and integral have decreased, implying the absorption of the electric field by the semiconductor material.

In conclusion, for the triangular geometry of the 100 nm base, there was an enhancement in terms of the electric field for all the distinct metals, as they all had wavelengths for which the electric field intensity was higher than that of the Silicon basic cell. In contrast, in terms of field integral, there was no improvement, as none of the different metal nanoantennas provided a larger field integral than the Si original cell, which might mean that for the triangular geometry of the 100 nm base, there are points of electric field concentration since higher values of electric field intensity are achieved, but this does not necessarily reflect a higher total electric field in the semiconductor.

**Figure 24:**
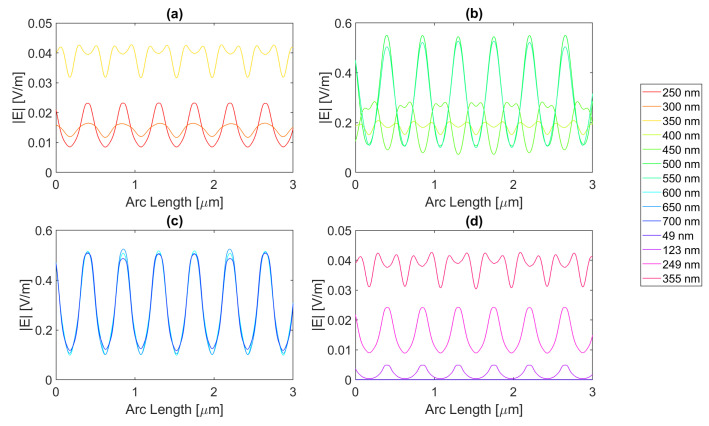
Electric field for the silver triangular nanostructure with a 200 nm base—Probe 1.

As for the simulations with the triangle base of 200 nm, there was electric field enhancement relative to the cell without nanoantennas for all metals, despite having no enhancement for the field integral. The simulated silver nanoantenna yields the electric field intensity curve seen in Figure 24. It has the maximum for a 500 nm wavelength of 5.551 × $10^{-1}$ V/m, with a field integral of 8.86 × $10^{-7}$. The results of the studied wavelengths can be found in Table A1, Table A2, and Table A3 for probes 1, 2, and 3, respectively. The electric field intensity increases from probe 1 to probe 2 for a range of wavelengths, from 650 to 700 nm, and the field integral increases for wavelengths between 600 and 700 nm. As for probe 3, both the electric field intensity and integral have decreased, indicating the absorption of the electric field by the semiconductor material.

**Figure 25:**
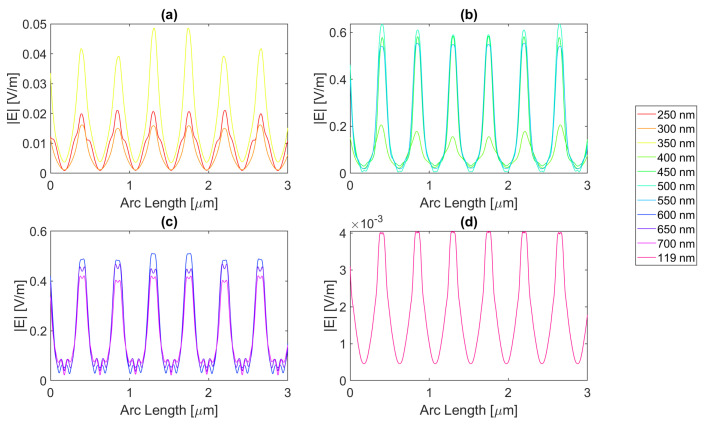
Electric field for the aluminium triangular nanostructure with a 200 nm base—Probe 1.

Figure 25 shows the electric field along probe 1 for the Aluminium antenna. For the distinct wavelengths, it shows that the maximum electric field is 6.36 × $10^{-1}$ V/m at 500 nm, with an integral of 5.78 × $10^{-7}$. The results of the studied wavelengths can be found in Table A4, Table A5, and Table A6 for probes 1, 2, and 3, respectively. The electric field intensity decreases from probe 1 to probe 2 for the whole range of wavelengths. As for the field integral, it increases for wavelengths between 500 and 700 nm. In probe 3, both the electric field intensity and integral have decreased, indicating the absorption of the electric field by the semiconductor material.

**Figure 26:**
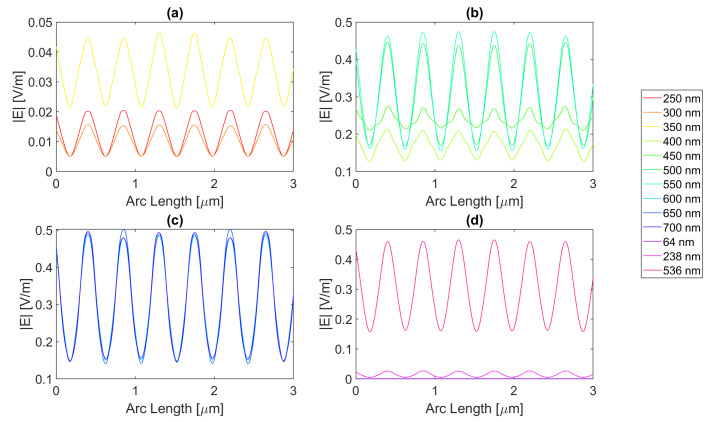
Electric field for the gold triangular nanostructure with a 200 nm base—Probe 1.

Regarding Figure 26, it is evident that the maximum electric field happens for a wavelength of 650 nm and is roughly 5.02 × $10^{-1}$ V/m. However, the highest field integral obtained is 9.20 × $10^{-7}$ for 600 nm. The results of the studied wavelengths can be found in Table A7, Table A8, and Table A9 for probes 1, 2, and 3, respectively. The electric field intensity increases from probe 1 to probe 2 for the wavelengths between 600 and 700 nm. As for the field integral, it increases for wavelengths between 500 and 700 nm. In probe 3, both the electric field intensity and integral have decreased, indicating the absorption of the electric field by the semiconductor material.

**Figure 27:**
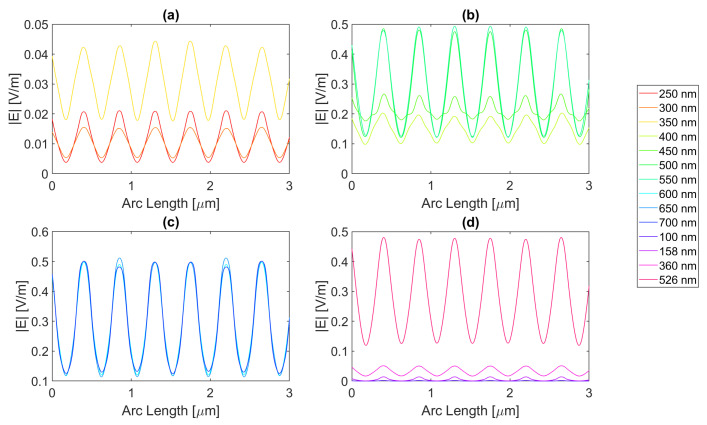
Electric field for the copper triangular nanostructure with a 200 nm base—Probe 1.

Lastly, Figure 27 illustrates the electric field at the first probe for copper, which peaks at 5.12 × $10^{-1}$ V/m for a wavelength of 650 nm, whereas the field integral peaks at 8.72 × $10^{-7}$ for a 700 nm wavelength. The results of the studied wavelengths can be found in Table A10, Table A11, and Table A12 for probes 1, 2, and 3, respectively. The electric field intensity increases from probe 1 to probe 2 for the wavelengths between 600 and 700 nm. As for the field integral, it decreases for all wavelengths. In probe 3, both the electric field intensity and integral have decreased, indicating the absorption of the electric field by the semiconductor material.

While this geometry yielded no improvements for the field integral, there were improvements in the electric field intensity for all combinations, meaning while this structure enhances and concentrates the electric field intensity in certain points, it does not mean it gives rise to a higher total field in the semiconductor.

## 5. Conclusions

From the sections above, key findings emerge; for instance, in Table A1, silver achieves the greatest $|E_{max}|$, 7.22 × $10^{-1}$ V/m at 550 nm, for the rectangular structure of 50 nm thickness. Additionally, the highest ∫Edx obtained is 1.48 × $10^{-6}$ for a circular structure with a 100 nm radius for a 700 nm wavelength. Overall, the silver structures in tandem with the Silicon cell yield an electric field intensity greater than that of the silicon cell alone 24 times.

Table A4 contains the results for aluminium. The highest $|E_{max}|$ achieved is 7.95 × $10^{-1}$ V/m for the rectangular structure of 50 nm thickness at 500 nm. Furthermore, it reaches its largest ∫Edx value, 1.43 × $10^{-6}$, for the circular structure with a 100 nm radius for 700 nm wavelength. Combining the results for all the Aluminium antennas, the silicon cell electric field intensity is surpassed a total of 21 times for this metal.

Concerning Table A7, which illustrates the results for gold, the antenna design that yields the greatest $|E_{max}|$ and ∫Edx is the circular antenna with a 100 nm radius at 700 nm with an electric field of 6.17 × $10^{-1}$ V/m and an ∫Edx of 1.42 × $10^{-6}$ at 700 nm. Now, compiling the results for the gold antenna structures, the electric intensity is greater than the silicon cell peak field intensity a total of 19 times.

Lastly, the antenna design giving rise to the highest values for copper, consulting Table A10, is the circular antenna with a 100 nm radius at 700 nm with an electric field of 6.49 × $10^{-1}$ V/m and an ∫Edx of 1.45 × $10^{-6}$ at 700 nm. Analysing the table results, one can observe that the electric field intensity surpasses the $|E_{max}{|}^{Si}$ 20 times. It can be concluded that the highest field concentration is for aluminium, with a field value of 7.95 × $10^{-1}$ V/m at 500 nm. As for the electric field integral, the highest value is reached for the silver circular antenna with a 100 nm radius.

For the rectangular nanoantenna, a higher field concentration is obtained for the 50 nm thick metal due to the fact that the added thickness facilitates the reflection of the electric field in the sides of the nanoantenna. Thus, resulting in a higher concentration of the electric field in the slit area.

Now analysing from a geometry standpoint, the 20 nm thick rectangular antenna has the best result when using silver with an electric field of 4.13 × $10^{-1}$ V/m at 550 nm, yet it is not an improvement compared with $|E_{max}{|}^{Si}$. In contrast, the 50-nm-thick rectangular antenna has a field enhancement for all the metals simulated, reaching its highest electric field of 7.95 × $10^{-1}$ for aluminium at 500 nm.

The circular geometry shows the electric field maxima for the same metal for both studied radii and the same wavelength. That is, for silver at 700 nm, the maximum electric field reached is 6.77 × $10^{-1}$ and 5.78 × $10^{-1}$ V/m for the 100 and 200 nm radius, respectively.

Finally, the triangular geometry delivers the electric field maxima for the different base lengths for the same metal, aluminium, but at distinct wavelengths. With a base length of 100 nm, the peak field is 5.08 × $10^{-1}$ V/m at 700 nm. As for the 200 nm base, at 500 nm, the maximum field achieved is 6.36 × $10^{-1}$ V/m. Notably, in a similar way to the circular geometry, the triangular geometries simulated all provide improvements for all considered metals for at least one of the frequencies when compared to $|E_{max}{|}^{Si}$.

It should also be noted that the geometry and metal used for the antenna not only influenced the electric field and its integral but also influenced the wavelengths for which it occurs. For example, for the 100 nm radius circular antenna, the maximum electric field intensity and integral all occurred for the same wavelength, at 700 nm, independently of the nanoantenna’s metal.

## Figures and Tables

**Table 1:** Drude–Lorentz parameters: Rakic’s fitting [14].

Parameters	Ag	Al	Au	Cu	
$\hbar \omega_{p}$	9.01	14.98	9.03	10.83	eV
$f_{0}$	0.845	0.523	0.760	0.575	
$\Gamma_{0}$	0.048	0.047	0.053	0.03	eV
$f_{1}$	0.065	0.227	0.024	0.061	
$\Gamma_{1}$	3.886	0.330	0.241	0.378	eV
$\omega_{1}$	0.816	0.162	0.415	0.291	eV
$f_{2}$	0.124	0.050	0.010	0.104	
$\Gamma_{2}$	0.452	0.312	0.345	1.056	eV
$\omega_{2}$	4.481	1.544	0.830	2.957	eV
$f_{3}$	0.011	0.166	0.071	0.723	
$\Gamma_{3}$	0.065	1.351	0.870	3.213	eV
$\omega_{3}$	8.185	1.808	2.969	5.3	eV
$f_{4}$	0.840	0.030	0.601	0.638	
$\Gamma_{4}$	0.916	3.382	2.494	4.305	eV
$\omega_{4}$	9.083	3.473	4.304	11.18	eV
$f_{5}$	5.646	-	4.384	-	
$\Gamma_{5}$	2.419	-	2.214	-	eV
$\omega_{5}$	20.29	-	13.32	-	eV

## Data Availability

Not applicable.

## Appendix A

**Table A1:** Summary table of the results obtained for the silver nanostructures—Probe 1.

		Rectangle Thickness = 20 nm		Rectangle Thickness = 50 nm		Circle r100		Circle r200		Triangle Base = 100 nm		Triangle Base = 200 nm	
	λ **[nm]**	$|E_{\max}|$ **[V/m]**	∫Edx	$|E_{\max}|$ **[V/m]**	∫Edx	$|E_{\max}|$ **[V/m]**	∫Edx	$|E_{\max}|$ **[V/m]**	∫Edx	$|E_{\max}|$ **[V/m]**	∫Edx	$|E_{\max}|$ **[V/m]**	∫Edx
UV	250	1.92 × $10^{-2}$	4.86 × $10^{-8}$	1.25 × $10^{-2}$	2.18 × $10^{-8}$	1.52 × $10^{-2}$	3.18 × $10^{-8}$	8.76 × $10^{-4}$	1.10 × $10^{-9}$	2.72 × $10^{-2}$	6.13 × $10^{-8}$	2.32 × $10^{-2}$	4.38 × $10^{-8}$
	300	1.70 × $10^{-2}$	4.68 × $10^{-8}$	1.46 × $10^{-2}$	3.35 × $10^{-8}$	1.78 × $10^{-2}$	3.27 × $10^{-8}$	2.82 × $10^{-3}$	6.02 × $10^{-9}$	1.76 × $10^{-2}$	4.78 × $10^{-8}$	1.65 × $10^{-2}$	4.34 × $10^{-8}$
	350	4.39 × $10^{-2}$	1.28 × $10^{-7}$	2.79 × $10^{-2}$	7.69 × $10^{-8}$	3.16 × $10^{-2}$	5.10 × $10^{-8}$	4.57 × $10^{-4}$	7.40 × $10^{-10}$	5.63 × $10^{-2}$	1.37 × $10^{-7}$	4.28 × $10^{-2}$	1.17 × $10^{-7}$
Visible Light	400	2.64 × $10^{-1}$	6.35 × $10^{-7}$	1.34 × $10^{-1}$	3.05 × $10^{-7}$	1.91 × $10^{-1}$	4.93 × $10^{-7}$	1.32 × $10^{-1}$	3.23 × $10^{-7}$	2.86 × $10^{-1}$	6.71 × $10^{-7}$	2.08 × $10^{-1}$	5.53 × $10^{-7}$
	450	3.47 × $10^{-1}$	8.26 × $10^{-7}$	4.68 × $10^{-1}$	5.97 × $10^{-7}$	2.67 × $10^{-1}$	5.91 × $10^{-7}$	1.09 × $10^{-1}$	2.64 × $10^{-7}$	3.77 × $10^{-1}$	9.13 × $10^{-7}$	2.85 × $10^{-1}$	6.18 × $10^{-7}$
	500	3.82 × $10^{-1}$	8.97 × $10^{-7}$	2.13 × $10^{-1}$	4.38 × $10^{-7}$	4.29 × $10^{-1}$	9.65 × $10^{-7}$	3.74 × $10^{-1}$	8.92 × $10^{-7}$	4.24 × $10^{-1}$	9.94 × $10^{-7}$	**5.51 × $10^{-1}$**	**8.86 × $10^{-7}$**
	550	**4.13 × $10^{-1}$**	9.25 × $10^{-7}$	**7.22 × $10^{-1}$**	**8.16 × $10^{-7}$**	4.63 × $10^{-1}$	1.08 × $10^{-6}$	4.34 × $10^{-1}$	9.81 × $10^{-7}$	4.43 × $10^{-1}$	1.04 × $10^{-6}$	5.27 × $10^{-1}$	8.70 × $10^{-7}$
	600	4.01 × $10^{-1}$	**9.29 × $10^{-7}$**	7.09 × $10^{-1}$	8.12 × $10^{-7}$	5.02 × $10^{-1}$	1.20 × $10^{-6}$	5.09 × $10^{-1}$	1.14 × $10^{-6}$	4.53 × $10^{-1}$	1.06 × $10^{-6}$	5.18 × $10^{-1}$	8.66 × $10^{-7}$
	650	3.77 × $10^{-1}$	9.08 × $10^{-7}$	5.83 × $10^{-1}$	6.50 × $10^{-7}$	6.18 × $10^{-1}$	1.41 × $10^{-6}$	5.66 × $10^{-1}$	1.37 × $10^{-6}$	4.59 × $10^{-1}$	1.07 × $10^{-6}$	5.25 × $10^{-1}$	8.64 × $10^{-7}$
	700	3.59 × $10^{-1}$	8.96 × $10^{-7}$	4.58 × $10^{-1}$	6.68 × $10^{-7}$	**6.77 × $10^{-1}$**	**1.48 × $10^{-6}$**	**5.78 × $10^{-1}$**	**1.40 × $10^{-6}$**	**4.71 × $10^{-1}$**	**1.09 × $10^{-6}$**	5.08 × $10^{-1}$	8.67 × $10^{-7}$
Resonance wavelengths	49	3.80 × $10^{-6}$	1.63 × $10^{-12}$	2.32 × $10^{-6}$	7.02 × $10^{-13}$	5.39 × $10^{-6}$	6.13 × $10^{-12}$	8.13 × $10^{-7}$	5.89 × $10^{-13}$	4.28 × $10^{-6}$	6.96 × $10^{-12}$	4.06 × $10^{-6}$	2.54 × $10^{-12}$
	123	3.47 × $10^{-3}$	5.85 × $10^{-9}$	1.85 × $10^{-3}$	1.61 × $10^{-9}$	7.08 × $10^{-3}$	6.82 × $10^{-9}$	6.21 × $10^{-5}$	6.87 × $10^{-11}$	6.51 × $10^{-3}$	1.00 × $10^{-8}$	4.93 × $10^{-3}$	5.95 × $10^{-9}$
	249	1.98 × $10^{-2}$	5.02 × $10^{-8}$	1.28 × $10^{-2}$	2.27 × $10^{-8}$	1.66 × $10^{-2}$	3.31 × $10^{-8}$	7.43 × $10^{-4}$	9.14 × $10^{-10}$	2.88 × $10^{-2}$	6.20 × $10^{-8}$	2.43 × $10^{-2}$	4.54 × $10^{-8}$
	355	4.40 × $10^{-2}$	1.26 × $10^{-7}$	2.69 × $10^{-2}$	7.39 × $10^{-8}$	3.38 × $10^{-2}$	5.85 × $10^{-8}$	1.19 × $10^{-3}$	2.24 × $10^{-9}$	5.65 × $10^{-2}$	1.36 × $10^{-7}$	4.27 × $10^{-2}$	1.15 × $10^{-7}$

**Table A2:** Summary table of the results obtained for the silver nanostructures—Probe 2.

		Rectangle Thickness = 20 nm		Rectangle Thickness = 50 nm		Circle r100		Circle r200		Triangle Base = 100 nm		Triangle Base = 200 nm	
	λ **[nm]**	$|E_{\max}|$ **[V/m]**	∫Edx	$|E_{\max}|$ **[V/m]**	∫Edx	$|E_{\max}|$ **[V/m]**	∫Edx	$|E_{\max}|$ **[V/m]**	∫Edx	$|E_{\max}|$ **[V/m]**	∫Edx	$|E_{\max}|$ **[V/m]**	∫Edx
UV	250	3.78 × $10^{-9}$	1.04 × $10^{-14}$	2.20 × $10^{-9}$	4.58 × $10^{-15}$	3.09 × $10^{-9}$	5.71 × $10^{-15}$	1.47 × $10^{-10}$	1.83 × $10^{-16}$	5.08 × $10^{-9}$	1.22 × $10^{-14}$	1.47 × $10^{-10}$	1.83 × $10^{-16}$
	300	6.83 × $10^{-9}$	1.98 × $10^{-14}$	5.49 × $10^{-9}$	1.41 × $10^{-14}$	7.13 × $10^{-9}$	1.34 × $10^{-14}$	1.05 × $10^{-9}$	2.32 × $10^{-15}$	7.25 × $10^{-9}$	2.01 × $10^{-14}$	1.05 × $10^{-9}$	2.32 × $10^{-15}$
	350	5.21 × $10^{-6}$	1.48 × $10^{-11}$	3.32 × $10^{-6}$	8.84 × $10^{-12}$	3.37 × $10^{-6}$	5.56 × $10^{-12}$	4.15 × $10^{-8}$	7.62 × $10^{-14}$	6.18 × $10^{-6}$	1.58 × $10^{-11}$	4.15 × $10^{-8}$	7.62 × $10^{-14}$
Visible Light	400	9.46 × $10^{-2}$	2.27 × $10^{-7}$	5.32 × $10^{-2}$	1.06 × $10^{-7}$	9.19 × $10^{-2}$	1.66 × $10^{-7}$	5.82 × $10^{-2}$	1.06 × $10^{-7}$	9.82 × $10^{-2}$	2.43 × $10^{-7}$	5.82 × $10^{-2}$	1.06 × $10^{-7}$
	450	2.29 × $10^{-1}$	5.83 × $10^{-7}$	2.40 × $10^{-1}$	4.45 × $10^{-7}$	2.17 × $10^{-1}$	3.96 × $10^{-7}$	9.55 × $10^{-2}$	1.74 × $10^{-7}$	2.68 × $10^{-1}$	6.30 × $10^{-7}$	9.55 × $10^{-2}$	1.74 × $10^{-7}$
	500	3.05 × $10^{-1}$	7.71 × $10^{-7}$	1.89 × $10^{-1}$	3.69 × $10^{-7}$	4.27 × $10^{-1}$	7.87 × $10^{-7}$	4.10 × $10^{-1}$	7.10 × $10^{-7}$	4.14 × $10^{-1}$	8.36 × $10^{-7}$	4.10 × $10^{-1}$	7.10 × $10^{-7}$
	550	3.57 × $10^{-1}$	8.55 × $10^{-7}$	4.93 × $10^{-1}$	8.82 × $10^{-7}$	4.32 × $10^{-1}$	9.73 × $10^{-7}$	4.24 × $10^{-1}$	8.70 × $10^{-7}$	4.87 × $10^{-1}$	9.35 × $10^{-7}$	4.24 × $10^{-1}$	8.70 × $10^{-7}$
	600	3.70 × $10^{-1}$	8.8 × $10^{-7}$	5.14 × $10^{-1}$	8.77 × $10^{-7}$	5.28 × $10^{-1}$	1.07 × $10^{-6}$	5.13 × $10^{-1}$	9.88 × $10^{-7}$	5.19 × $10^{-1}$	9.76 × $10^{-7}$	5.13 × $10^{-1}$	9.88 × $10^{-7}$
	650	3.47 × $10^{-1}$	8.8 × $10^{-7}$	3.81 × $10^{-1}$	7.15 × $10^{-7}$	6.25 × $10^{-1}$	1.31 × $10^{-6}$	5.66 × $10^{-1}$	1.22 × $10^{-6}$	5.71 × $10^{-1}$	1.00 × $10^{-6}$	5.66 × $10^{-1}$	1.22 × $10^{-6}$
	700	3.28 × $10^{-1}$	8.59 × $10^{-7}$	2.64 × $10^{-1}$	5.66 × $10^{-7}$	6.55 × $10^{-1}$	1.43 × $10^{-6}$	6.18 × $10^{-1}$	1.28 × $10^{-6}$	5.84 × $10^{-1}$	1.02 × $10^{-6}$	6.18 × $10^{-1}$	1.28 × $10^{-6}$
Resonance wavelengths	49	1.37 × $10^{-33}$	8.47 × $10^{-40}$	6.35 × $10^{-34}$	3.04 × $10^{-40}$	2.60 × $10^{-33}$	3.27 × $10^{-39}$	3.62 × $10^{-34}$	2.74 × $10^{-40}$	2.30 × $10^{-33}$	3.81 × $10^{-39}$	3.62 × $10^{-34}$	2.74 × $10^{-40}$
	123	4.00 × $10^{-14}$	8.12 × $10^{-20}$	1.92 × $10^{-14}$	2.04 × $10^{-20}$	8.30 × $10^{-14}$	9.22 × $10^{-20}$	5.45 × $10^{-16}$	6.92 × $10^{-22}$	7.57 × $10^{-14}$	1.40 × $10^{-19}$	5.45 × $10^{-16}$	6.92 × $10^{-22}$
	249	4.27 × $10^{-9}$	1.17 × $10^{-14}$	2.48 × $10^{-9}$	5.23 × $10^{-15}$	3.69 × $10^{-9}$	6.59 × $10^{-15}$	1.36 × $10^{-10}$	1.68 × $10^{-16}$	5.64 × $10^{-9}$	1.35 × $10^{-14}$	1.36 × $10^{-10}$	1.68 × $10^{-16}$
	355	5.82 × $10^{-6}$	1.61 × $10^{-11}$	3.54 × $10^{-6}$	9.40 × $10^{-12}$	4.00 × $10^{-6}$	6.96 × $10^{-12}$	1.20 × $10^{-7}$	2.52 × $10^{-13}$	6.84 × $10^{-6}$	1.74 × $10^{-11}$	1.20 × $10^{-7}$	2.52 × $10^{-13}$

**Table A3:** Summary table of the results obtained for the silver nanostructures—Probe 3.

		Rectangle Thickness = 20 nm		Rectangle Thickness = 50 nm		Circle r100		Circle r200		Triangle Base = 100 nm		Triangle Base = 200 nm	
	λ **[nm]**	$|E_{\max}|$ **[V/m]**	∫Edx	$|E_{\max}|$ **[V/m]**	∫Edx	$|E_{\max}|$ **[V/m]**	∫Edx	$|E_{\max}|$ **[V/m]**	∫Edx	$|E_{\max}|$ **[V/m]**	∫Edx	$|E_{\max}|$ **[V/m]**	∫Edx
UV	250	8.46 × $10^{-61}$	1.86 × $10^{-66}$	3.86 × $10^{-61}$	8.11 × $10^{-67}$	4.22 × $10^{-61}$	8.19 × $10^{-67}$	1.44 × $10^{-62}$	2.53 × $10^{-68}$	9.95 × $10^{-61}$	2.11 × $10^{-66}$	7.71 × $10^{-61}$	1.73 × $10^{-66}$
	300	5.37 × $10^{-58}$	1.23 × $10^{-63}$	3.55 × $10^{-58}$	8.39 × $10^{-64}$	3.54 × $10^{-58}$	7.41 × $10^{-64}$	4.92 × $10^{-59}$	1.01 × $10^{-64}$	5.43 × $10^{-58}$	1.25 × $10^{-63}$	4.78 × $10^{-58}$	1.12 × $10^{-63}$
	350	3.37 × $10^{-36}$	8.79 × $10^{-42}$	1.91 × $10^{-36}$	5.03 × $10^{-42}$	1.25 × $10^{-36}$	2.63 × $10^{-42}$	1.35 × $10^{-38}$	2.75 × $10^{-44}$	3.41 × $10^{-36}$	9.33 × $10^{-42}$	3.08 × $10^{-36}$	7.83 × $10^{-42}$
Visible Light	400	4.19 × $10^{-5}$	1.06 × $10^{-10}$	2.22 × $10^{-5}$	4.74 × $10^{-11}$	3.60 × $10^{-5}$	6.41 × $10^{-11}$	1.73 × $10^{-5}$	3.79 × $10^{-11}$	4.59 × $10^{-5}$	1.12 × $10^{-10}$	3.61 × $10^{-5}$	9.24 × $10^{-11}$
	450	1.68 × $10^{-2}$	4.49 × $10^{-8}$	1.51 × $10^{-2}$	3.32 × $10^{-8}$	1.98 × $10^{-2}$	2.36 × $10^{-8}$	8.87 × $10^{-3}$	8.28 × $10^{-9}$	2.50 × $10^{-2}$	4.56 × $10^{-8}$	1.86 × $10^{-2}$	3.12 × $10^{-8}$
	500	1.01 × $10^{-1}$	2.68 × $10^{-7}$	6.43 × $10^{-2}$	1.23 × $10^{-7}$	1.17 × $10^{-1}$	2.64 × $10^{-7}$	1.31 × $10^{-1}$	2.20 × $10^{-7}$	1.21 × $10^{-1}$	3.11 × $10^{-7}$	1.37 × $10^{-1}$	2.97 × $10^{-7}$
	550	2.05 × $10^{-1}$	5.01 × $10^{-7}$	2.97 × $10^{-1}$	4.45 × $10^{-7}$	2.22 × $10^{-1}$	5.63 × $10^{-7}$	2.08 × $10^{-1}$	4.92 × $10^{-7}$	2.63 × $10^{-1}$	5.79 × $10^{-7}$	2.94 × $10^{-1}$	4.94 × $10^{-7}$
	600	2.47 × $10^{-1}$	6.24 × $10^{-7}$	2.82 × $10^{-1}$	6.10 × $10^{-7}$	3.08 × $10^{-1}$	7.78 × $10^{-7}$	3.29 × $10^{-1}$	6.80 × $10^{-7}$	3.92 × $10^{-1}$	7.23 × $10^{-7}$	3.30 × $10^{-1}$	6.16 × $10^{-7}$
	650	3.09 × $10^{-1}$	7.36 × $10^{-7}$	3.47 × $10^{-1}$	5.42 × $10^{-7}$	5.24 × $10^{-1}$	1.10 × $10^{-6}$	4.32 × $10^{-1}$	1.03 × $10^{-6}$	4.01 × $10^{-1}$	9.01 × $10^{-7}$	4.03 × $10^{-1}$	7.70 × $10^{-7}$
	700	3.10 × $10^{-1}$	7.63 × $10^{-7}$	2.38 × $10^{-1}$	4.95 × $10^{-7}$	6.82 × $10^{-1}$	1.23 × $10^{-6}$	6.15 × $10^{-1}$	1.11 × $10^{-6}$	4.96 × $10^{-1}$	9.87 × $10^{-7}$	4.24 × $10^{-1}$	8.45 × $10^{-7}$
Resonance wavelengths	49	0	0	0	0	0	0	0	0	0	0	0	0
	123	1.25 × $10^{-98}$	1 × $10^{-104}$	3.89 × $10^{-99}$	2.65 × $10^{-105}$	1.04 × $10^{-98}$	1.08 × $10^{-104}$	8.74 × $10^{-101}$	6.12 × $10^{-107}$	3.15 × $10^{-98}$	1.84 × $10^{-104}$	1.46 × $10^{-98}$	1.22 × $10^{-104}$
	249	1.90 × $10^{-60}$	4.2 × $10^{-66}$	8.75 × $10^{-61}$	1.84 × $10^{-66}$	9.86 × $10^{-61}$	1.91 × $10^{-66}$	2.65 × $10^{-62}$	4.69 × $10^{-68}$	2.20 × $10^{-60}$	4.70 × $10^{-66}$	1.73 × $10^{-60}$	3.91 × $10^{-66}$
	355	8.84 × $10^{-36}$	2.29 × $10^{-41}$	4.85 × $10^{-36}$	1.27 × $10^{-41}$	3.52 × $10^{-36}$	7.44 × $10^{-42}$	1.05 × $10^{-37}$	2.12 × $10^{-43}$	9.32 × $10^{-36}$	2.45 × $10^{-41}$	8.20 × $10^{-36}$	2.01 × $10^{-41}$

**Table A4:** Summary table of the results obtained for the aluminium nanostructures—Probe 1.

		Rectangle Thickness = 20 nm		Rectangle Thickness = 50 nm		Circle r100		Circle r200		Triangle Base = 100 nm		Triangle Base = 200 nm	
	λ **[nm]**	$|E_{\max}|$ **[V/m]**	∫Edx	$|E_{\max}|$ **[V/m]**	∫Edx	$|E_{\max}|$ **[V/m]**	∫Edx	$|E_{\max}|$ **[V/m]**	∫Edx	$|E_{\max}|$ **[V/m]**	∫Edx	$|E_{\max}|$ **[V/m]**	∫Edx
UV	250	2.55 × $10^{-2}$	3.34 × $10^{-8}$	8.54 × $10^{-3}$	5.76 × $10^{-9}$	2.95 × $10^{-2}$	5.97 × $10^{-8}$	2.44 × $10^{-2}$	4.71 × $10^{-8}$	5.07 × $10^{-2}$	6.47 × $10^{-8}$	2.11 × $10^{-2}$	2.68 × $10^{-8}$
	300	2.36 × $10^{-2}$	2.45 × $10^{-8}$	2.25 × $10^{-2}$	1.28 × $10^{-8}$	2.71 × $10^{-2}$	4.05 × $10^{-8}$	1.41 × $10^{-2}$	3.42 × $10^{-8}$	2.20 × $10^{-2}$	3.78 × $10^{-8}$	1.62 × $10^{-2}$	1.93 × $10^{-8}$
	350	5.86 × $10^{-2}$	6.83 × $10^{-8}$	7.09 × $10^{-2}$	4.05 × $10^{-8}$	6.37 × $10^{-2}$	1.40 × $10^{-7}$	4.49 × $10^{-2}$	1.07 × $10^{-7}$	5.98 × $10^{-2}$	1.07 × $10^{-7}$	4.86 × $10^{-2}$	5.32 × $10^{-8}$
Visible Light	400	2.84 × $10^{-1}$	3.47 × $10^{-7}$	2.87 × $10^{-1}$	1.72 × $10^{-7}$	3.41 × $10^{-1}$	6.97 × $10^{-7}$	1.88 × $10^{-1}$	4.47 × $10^{-7}$	3.02 × $10^{-1}$	5.17 × $10^{-7}$	2.06 × $10^{-1}$	2.43 × $10^{-7}$
	450	2.44 × $10^{-1}$	3.95 × $10^{-7}$	1.35 × $10^{-1}$	1.14 × $10^{-7}$	3.91 × $10^{-1}$	8.28 × $10^{-7}$	3.29 × $10^{-1}$	7.64 × $10^{-7}$	4.65 × $10^{-1}$	8.01 × $10^{-7}$	5.84 × $10^{-1}$	5.31 × $10^{-7}$
	500	**3.47 × $10^{-1}$**	4.56 × $10^{-7}$	**7.95 × $10^{-1}$**	7.22 × $10^{-7}$	4.85 × $10^{-1}$	1.06 × $10^{-6}$	3.73 × $10^{-1}$	9.17 × $10^{-7}$	4.72 × $10^{-1}$	8.51 × $10^{-7}$	**6.36 × $10^{-1}$**	**5.78 × $10^{-7}$**
	550	3.38 × $10^{-1}$	4.89 × $10^{-7}$	5.40 × $10^{-1}$	6.44 × $10^{-7}$	5.27 × $10^{-1}$	1.20 × $10^{-6}$	3.99 × $10^{-1}$	1.07 × $10^{-6}$	4.72 × $10^{-1}$	8.68 × $10^{-7}$	5.53 × $10^{-1}$	5.55 × $10^{-7}$
	600	3.31 × $10^{-1}$	5.75 × $10^{-7}$	4.47 × $10^{-1}$	**7.74 × $10^{-7}$**	5.43 × $10^{-1}$	1.31 × $10^{-6}$	**4.49 × $10^{-1}$**	**1.20 × $10^{-6}$**	5.05 × $10^{-1}$	8.84 × $10^{-7}$	5.11 × $10^{-1}$	5.65 × $10^{-7}$
	650	3.02 × $10^{-1}$	**5.97 × $10^{-7}$**	3.89 × $10^{-1}$	7.42 × $10^{-7}$	5.62 × $10^{-1}$	1.38 × $10^{-6}$	4.28 × $10^{-1}$	1.16 × $10^{-6}$	**5.08 × $10^{-1}$**	8.84 × $10^{-7}$	4.69 × $10^{-1}$	5.63 × $10^{-7}$
	700	3.18 × $10^{-1}$	5.58 × $10^{-7}$	3.80 × $10^{-1}$	6.48 × $10^{-7}$	**5.78 × $10^{-1}$**	**1.43 × $10^{-6}$**	3.90 × $10^{-1}$	1.03 × $10^{-6}$	4.91 × $10^{-1}$	**9.14 × $10^{-7}$**	4.21 × $10^{-1}$	5.24 × $10^{-7}$
Resonance wavelengths	119	2.58 × $10^{-3}$	5.48 × $10^{-9}$	3.48 × $10^{-4}$	8.53 × $10^{-10}$	8.35 × $10^{-3}$	7.46 × $10^{-9}$	4.51 × $10^{-7}$	3.27 × $10^{-13}$	5.35 × $10^{-3}$	8.89 × $10^{-9}$	4.05 × $10^{-3}$	5.42 × $10^{-9}$

**Table A5:** Summary table of the results obtained for the aluminium nanostructures—Probe 2.

		Rectangle Thickness = 20 nm		Rectangle Thickness = 50 nm		Circle r100		Circle r200		Triangle Base = 100 nm		Triangle Base = 200 nm	
	λ **[nm]**	$|E_{\max}|$ **[V/m]**	∫Edx	$|E_{\max}|$ **[V/m]**	∫Edx	$|E_{\max}|$ **[V/m]**	∫Edx	$|E_{\max}|$ **[V/m]**	∫Edx	$|E_{\max}|$ **[V/m]**	∫Edx	$|E_{\max}|$ **[V/m]**	∫Edx
UV	250	3.05 × $10^{-9}$	5.17 × $10^{-15}$	1.07 × $10^{-9}$	9.33 × $10^{-16}$	5.32 × $10^{-9}$	9.63 × $10^{-15}$	4.16 × $10^{-9}$	6.34 × $10^{-15}$	7.42 × $10^{-9}$	1.29 × $10^{-14}$	3.76 × $10^{-9}$	5.13 × $10^{-15}$
	300	7.25 × $10^{-9}$	1.02 × $10^{-14}$	6.37 × $10^{-9}$	5.44 × $10^{-15}$	1.05 × $10^{-8}$	1.57 × $10^{-14}$	4.71 × $10^{-9}$	1.10 × $10^{-14}$	7.78 × $10^{-9}$	1.58 × $10^{-14}$	6.66 × $10^{-9}$	7.82 × $10^{-15}$
	350	4.77 × $10^{-6}$	7.83 × $10^{-12}$	5.14 × $10^{-6}$	5.04 × $10^{-12}$	7.10 × $10^{-6}$	1.43 × $10^{-11}$	5.16 × $10^{-6}$	9.92 × $10^{-12}$	5.98 × $10^{-6}$	1.22 × $10^{-11}$	5.42 × $10^{-6}$	5.84 × $10^{-12}$
Visible Light	400	7.03 × $10^{-2}$	1.28 × $10^{-7}$	6.06 × $10^{-2}$	8.68 × $10^{-8}$	1.37 × $10^{-1}$	2.36 × $10^{-7}$	8.92 × $10^{-2}$	1.47 × $10^{-7}$	9.72 × $10^{-2}$	1.86 × $10^{-7}$	7.84 × $10^{-2}$	8.26 × $10^{-8}$
	450	1.20 × $10^{-1}$	2.80 × $10^{-7}$	5.47 × $10^{-2}$	9.88 × $10^{-8}$	2.87 × $10^{-1}$	5.53 × $10^{-7}$	2.63 × $10^{-1}$	5.10 × $10^{-7}$	4.19 × $10^{-1}$	5.15 × $10^{-7}$	3.65 × $10^{-1}$	4.02 × $10^{-7}$
	500	1.90 × $10^{-1}$	3.80 × $10^{-7}$	3.37 × $10^{-1}$	7.27 × $10^{-7}$	4.28 × $10^{-1}$	8.88 × $10^{-7}$	3.64 × $10^{-1}$	7.53 × $10^{-7}$	5.11 × $10^{-1}$	7.32 × $10^{-7}$	4.56 × $10^{-1}$	5.88 × $10^{-7}$
	550	1.93 × $10^{-1}$	3.98 × $10^{-7}$	2.21 × $10^{-1}$	4.85 × $10^{-7}$	5.13 × $10^{-1}$	1.07 × $10^{-6}$	4.23 × $10^{-1}$	9.61 × $10^{-7}$	5.45 × $10^{-1}$	8.12 × $10^{-7}$	4.18 × $10^{-1}$	6.31 × $10^{-7}$
	600	2.21 × $10^{-1}$	4.21 × $10^{-7}$	2.48 × $10^{-1}$	5.05 × $10^{-7}$	5.96 × $10^{-1}$	1.19 × $10^{-6}$	5.39 × $10^{-1}$	1.06 × $10^{-6}$	5.52 × $10^{-1}$	8.18 × $10^{-7}$	4.07 × $10^{-1}$	6.16 × $10^{-7}$
	650	1.76 × $10^{-1}$	3.86 × $10^{-7}$	1.36 × $10^{-1}$	3.16 × $10^{-7}$	6.13 × $10^{-1}$	1.31 × $10^{-6}$	5.17 × $10^{-1}$	1.07 × $10^{-6}$	5.46 × $10^{-1}$	8.46 × $10^{-7}$	3.57 × $10^{-1}$	6.02 × $10^{-7}$
	700	1.67 × $10^{-1}$	3.92 × $10^{-7}$	1.62 × $10^{-1}$	4.04 × $10^{-7}$	6.15 × $10^{-1}$	1.38 × $10^{-6}$	4.44 × $10^{-1}$	9.74 × $10^{-7}$	5.37 × $10^{-1}$	8.90 × $10^{-7}$	3.10 × $10^{-1}$	5.67 × $10^{-7}$
Resonance wavelengths	119	1.32 × $10^{-14}$	3.23 × $10^{-20}$	2.08 × $10^{-15}$	4.80 × $10^{-21}$	3.78 × $10^{-14}$	3.81 × $10^{-20}$	1.71 × $10^{-18}$	1.50 × $10^{-24}$	2.73 × $10^{-14}$	5.28 × $10^{-20}$	2.30 × $10^{-14}$	3.16 × $10^{-20}$

**Table A6:** Summary table of the results obtained for the aluminium nanostructures—Probe 3.

		Rectangle Thickness = 20 nm		Rectangle Thickness = 50 nm		Circle r100		Circle r200		Triangle Base = 100 nm		Triangle Base = 200 nm	
	λ **[nm]**	$|E_{\max}|$ **[V/m]**	∫Edx	$|E_{\max}|$ **[V/m]**	∫Edx	$|E_{\max}|$ **[V/m]**	∫Edx	$|E_{\max}|$ **[V/m]**	∫Edx	$|E_{\max}|$ **[V/m]**	∫Edx	$|E_{\max}|$ **[V/m]**	∫Edx
UV	250	3.02 × $10^{-61}$	6.51 × $10^{-67}$	7.53 × $10^{-62}$	8.97 × $10^{-68}$	7.09 × $10^{-61}$	1.52 × $10^{-66}$	3.97 × $10^{-61}$	6.62 × $10^{-67}$	1.06 × $10^{-60}$	2.26 × $10^{-66}$	4.43 × $10^{-61}$	8.60 × $10^{-67}$
	300	3.22 × $10^{-58}$	6.21 × $10^{-64}$	1.73 × $10^{-58}$	2.84 × $10^{-64}$	4.70 × $10^{-58}$	7.76 × $10^{-64}$	1.82 × $10^{-58}$	3.29 × $10^{-64}$	5.12 × $10^{-58}$	9.57 × $10^{-64}$	2.86 × $10^{-58}$	4.80 × $10^{-64}$
	350	2.02 × $10^{-36}$	4.58 × $10^{-42}$	1.31 × $10^{-36}$	2.63 × $10^{-42}$	3.18 × $10^{-36}$	5.69 × $10^{-42}$	1.97 × $10^{-36}$	3.25 × $10^{-42}$	3.22 × $10^{-36}$	7.02 × $10^{-42}$	1.82 × $10^{-36}$	3.52 × $10^{-42}$
Visible Light	400	2.76 × $10^{-5}$	5.75 × $10^{-11}$	1.93 × $10^{-5}$	3.36 × $10^{-11}$	5.74 × $10^{-5}$	9.13 × $10^{-11}$	3.05 × $10^{-5}$	5.60 × $10^{-11}$	3.98 × $10^{-5}$	8.80 × $10^{-11}$	2.41 × $10^{-5}$	4.38 × $10^{-11}$
	450	9.26 × $10^{-3}$	2.08 × $10^{-8}$	3.05 × $10^{-3}$	6.58 × $10^{-9}$	2.59 × $10^{-2}$	3.49 × $10^{-8}$	2.47 × $10^{-2}$	2.78 × $10^{-8}$	2.04 × $10^{-2}$	4.75 × $10^{-8}$	1.57 × $10^{-2}$	3.73 × $10^{-8}$
	500	6.18 × $10^{-2}$	1.29 × $10^{-7}$	1.38 × $10^{-1}$	1.86 × $10^{-7}$	1.33 × $10^{-1}$	2.94 × $10^{-7}$	1.16 × $10^{-1}$	2.26 × $10^{-7}$	1.32 × $10^{-1}$	2.87 × $10^{-7}$	1.48 × $10^{-1}$	2.24 × $10^{-7}$
	550	1.14 × $10^{-1}$	2.15 × $10^{-7}$	1.37 × $10^{-1}$	2.24 × $10^{-7}$	2.71 × $10^{-1}$	6.33 × $10^{-7}$	2.09 × $10^{-1}$	5.39 × $10^{-7}$	3.07 × $10^{-1}$	4.97 × $10^{-7}$	2.58 × $10^{-1}$	3.57 × $10^{-7}$
	600	1.35 × $10^{-1}$	2.84 × $10^{-7}$	1.43 × $10^{-1}$	3.24 × $10^{-7}$	3.41 × $10^{-1}$	8.81 × $10^{-7}$	3.61 × $10^{-1}$	7.57 × $10^{-7}$	3.78 × $10^{-1}$	6.23 × $10^{-7}$	2.25 × $10^{-1}$	4.87 × $10^{-7}$
	650	1.66 × $10^{-1}$	3.02 × $10^{-7}$	1.36 × $10^{-1}$	2.25 × $10^{-7}$	5.29 × $10^{-1}$	1.11 × $10^{-6}$	3.94 × $10^{-1}$	9.18 × $10^{-7}$	3.88 × $10^{-1}$	8.12 × $10^{-7}$	3.00 × $10^{-1}$	5.36 × $10^{-7}$
	700	1.69 × $10^{-1}$	3.21 × $10^{-7}$	1.43 × $10^{-1}$	3.40 × $10^{-7}$	6.18 × $10^{-1}$	1.22 × $10^{-6}$	4.84 × $10^{-1}$	8.35 × $10^{-7}$	4.22 × $10^{-1}$	9.04 × $10^{-7}$	2.50 × $10^{-1}$	5.59 × $10^{-7}$
Resonance wavelengths	119	5.68 × $10^{-102}$	4.00 × $10^{-108}$	1.42 × $10^{-102}$	7.57 × $10^{-109}$	4.55 × $10^{-102}$	3.80 × $10^{-108}$	1.88 × $10^{-106}$	1.21 × $10^{-112}$	1.52 × $10^{-101}$	7.21 × $10^{-108}$	6.25 × $10^{-102}$	4.40 × $10^{-108}$

**Table A7:** Summary table of the results obtained for the gold nanostructures—Probe 1.

		Rectangle, Height 20 nm		Rectangle, Height 50 nm		Circle, Radius 100 nm		Circle, Radius 200 nm		Triangle, Base 100 nm		Triangle, Base 200 nm	
	λ **[nm]**	$|E_{\max}|$ **[V/m]**	∫Edx	$|E_{\max}|$ **[V/m]**	∫Edx	$|E_{\max}|$ **[V/m]**	∫Edx	$|E_{\max}|$ **[V/m]**	∫Edx	$|E_{\max}|$ **[V/m]**	∫Edx	$|E_{\max}|$ **[V/m]**	∫Edx
UV	250	2.17 × $10^{-2}$	4.33 × $10^{-8}$	1.33 × $10^{-2}$	1.72 × $10^{-8}$	1.70 × $10^{-2}$	3.80 × $10^{-8}$	4.19 × $10^{-3}$	6.47 × $10^{-9}$	3.46 × $10^{-2}$	6.12 × $10^{-8}$	2.04 × $10^{-2}$	3.76 × $10^{-8}$
	300	1.81 × $10^{-2}$	3.55 × $10^{-8}$	1.39 × $10^{-2}$	1.71 × $10^{-8}$	1.94 × $10^{-2}$	3.17 × $10^{-8}$	3.59 × $10^{-3}$	7.76 × $10^{-9}$	1.93 × $10^{-2}$	4.25 × $10^{-8}$	1.57 × $10^{-2}$	3.02 × $10^{-8}$
	350	5.11 × $10^{-2}$	1.15 × $10^{-7}$	4.38 × $10^{-2}$	6.40 × $10^{-8}$	5.04 × $10^{-2}$	1.05 × $10^{-7}$	9.73 × $10^{-3}$	2.29 × $10^{-8}$	5.32 × $10^{-2}$	1.29 × $10^{-7}$	4.64 × $10^{-2}$	9.93 × $10^{-8}$
Visible Light	400	2.51 × $10^{-1}$	5.85 × $10^{-7}$	2.25 × $10^{-1}$	3.52 × $10^{-7}$	1.98 × $10^{-1}$	4.86 × $10^{-7}$	4.79 × $10^{-2}$	1.24 × $10^{-7}$	2.58 × $10^{-1}$	6.45 × $10^{-7}$	2.14 × $10^{-1}$	5.08 × $10^{-7}$
	450	3.10 × $10^{-1}$	7.96 × $10^{-7}$	2.65 × $10^{-1}$	5.15 × $10^{-7}$	1.97 × $10^{-1}$	5.00 × $10^{-7}$	4.68 × $10^{-2}$	1.20 × $10^{-7}$	3.51 × $10^{-1}$	8.82 × $10^{-7}$	2.75 × $10^{-1}$	7.12 × $10^{-7}$
	500	3.50 × $10^{-1}$	9.10 × $10^{-7}$	2.32 × $10^{-1}$	5.59 × $10^{-7}$	2.55 × $10^{-1}$	6.89 × $10^{-7}$	7.84 × $10^{-2}$	2.12 × $10^{-7}$	4.03 × $10^{-1}$	1.00 × $10^{-6}$	4.46 × $10^{-1}$	8.77 × $10^{-7}$
	550	3.91 × $10^{-1}$	9.67 × $10^{-7}$	3.39 × $10^{-1}$	6.28 × $10^{-7}$	3.73 × $10^{-1}$	9.32 × $10^{-7}$	2.12 × $10^{-1}$	5.67 × $10^{-7}$	4.26 × $10^{-1}$	1.06 × $10^{-6}$	4.74 × $10^{-1}$	9.15 × $10^{-7}$
	600	**4.00 × $10^{-1}$**	**9.87 × $10^{-7}$**	5.67 × $10^{-1}$	**8.02 × $10^{-7}$**	4.61 × $10^{-1}$	1.12 × $10^{-6}$	3.58 × $10^{-1}$	8.59 × $10^{-7}$	4.42 × $10^{-1}$	1.08 × $10^{-6}$	4.87 × $10^{-1}$	**9.20 × $10^{-7}$**
	650	3.99 × $10^{-1}$	9.85 × $10^{-7}$	**5.94 × $10^{-1}$**	7.69 × $10^{-7}$	5.21 × $10^{-1}$	1.27 × $10^{-6}$	4.62 × $10^{-1}$	1.10 × $10^{-6}$	4.50 × $10^{-1}$	1.10 × $10^{-6}$	**5.02 × $10^{-1}$**	9.18 × $10^{-7}$
	700	3.80 × $10^{-1}$	9.67 × $10^{-7}$	5.12 × $10^{-1}$	6.96 × $10^{-7}$	**6.17 × $10^{-1}$**	**1.42 × $10^{-6}$**	**5.46 × $10^{-1}$**	**1.29 × $10^{-6}$**	**4.58 × $10^{-1}$**	**1.11 × $10^{-6}$**	4.97 × $10^{-1}$	9.17 × $10^{-7}$
Resonance wavelengths	64	5.29 × $10^{-5}$	2.93 × $10^{-11}$	3.40 × $10^{-5}$	1.50 × $10^{-11}$	7.02 × $10^{-5}$	1.03 × $10^{-10}$	5.80 × $10^{-6}$	7.13 × $10^{-12}$	6.68 × $10^{-5}$	1.14 × $10^{-10}$	6.19 × $10^{-5}$	4.05 × $10^{-11}$
	238	2.36 × $10^{-2}$	4.78 × $10^{-8}$	1.58 × $10^{-2}$	1.91 × $10^{-8}$	2.15 × $10^{-2}$	4.84 × $10^{-8}$	4.32 × $10^{-3}$	6.22 × $10^{-9}$	3.40 × $10^{-2}$	6.92 × $10^{-8}$	2.65 × $10^{-2}$	4.35 × $10^{-8}$
	536	3.81 × $10^{-1}$	9.54 × $10^{-7}$	2.74 × $10^{-1}$	5.87 × $10^{-7}$	3.34 × $10^{-1}$	8.65 × $10^{-7}$	1.73 × $10^{-1}$	4.62 × $10^{-7}$	4.22 × $10^{-1}$	1.04 × $10^{-6}$	4.65 × $10^{-1}$	9.06 × $10^{-7}$

**Table A8:** Summary table of the results obtained for the gold nanostructures—Probe 2.

		Rectangle, Height 20 nm		Rectangle, Height 50 nm		Circle, Radius 100 nm		Circle, Radius 200 nm		Triangle, Base 100 nm		Triangle, Base 200 nm	
	λ **[nm]**	$|E_{\max}|$ **[V/m]**	∫Edx	$|E_{\max}|$ **[V/m]**	∫Edx	$|E_{\max}|$ **[V/m]**	∫Edx	$|E_{\max}|$ **[V/m]**	∫Edx	$|E_{\max}|$ **[V/m]**	∫Edx	$|E_{\max}|$ **[V/m]**	∫Edx
UV	250	3.86 × $10^{-9}$	9.20 × $10^{-15}$	2.10 × $10^{-9}$	3.64 × $10^{-15}$	3.58 × $10^{-9}$	7.07 × $10^{-15}$	7.25 × $10^{-10}$	1.04 × $10^{-15}$	5.87 × $10^{-9}$	1.27 × $10^{-14}$	4.09 × $10^{-9}$	8.06 × $10^{-15}$
	300	6.37 × $10^{-9}$	1.50 × $10^{-14}$	4.52 × $10^{-9}$	7.20 × $10^{-15}$	7.65 × $10^{-9}$	1.27 × $10^{-14}$	1.29 × $10^{-9}$	2.82 × $10^{-15}$	7.37 × $10^{-9}$	1.79 × $10^{-14}$	6.40 × $10^{-9}$	1.28 × $10^{-14}$
	350	5.05 × $10^{-6}$	1.33 × $10^{-11}$	3.90 × $10^{-6}$	7.42 × $10^{-12}$	5.38 × $10^{-6}$	1.14 × $10^{-11}$	9.54 × $10^{-7}$	2.37 × $10^{-12}$	5.81 × $10^{-6}$	1.49 × $10^{-11}$	5.22 × $10^{-6}$	1.15 × $10^{-11}$
Visible Light	400	7.82 × $10^{-2}$	2.12 × $10^{-7}$	6.21 × $10^{-2}$	1.30 × $10^{-7}$	7.66 × $10^{-2}$	1.71 × $10^{-7}$	1.90 × $10^{-2}$	4.35 × $10^{-8}$	8.94 × $10^{-2}$	2.33 × $10^{-7}$	7.77 × $10^{-2}$	1.84 × $10^{-7}$
	450	2.00 × $10^{-1}$	5.63 × $10^{-7}$	1.48 × $10^{-1}$	3.68 × $10^{-7}$	1.49 × $10^{-1}$	3.46 × $10^{-7}$	3.63 × $10^{-2}$	8.35 × $10^{-8}$	2.59 × $10^{-1}$	6.19 × $10^{-7}$	2.04 × $10^{-1}$	5.01 × $10^{-7}$
	500	2.83 × $10^{-1}$	7.85 × $10^{-7}$	2.04 × $10^{-1}$	4.82 × $10^{-7}$	2.72 × $10^{-1}$	5.75 × $10^{-7}$	8.56 × $10^{-2}$	1.76 × $10^{-7}$	3.77 × $10^{-1}$	8.59 × $10^{-7}$	4.06 × $10^{-1}$	7.39 × $10^{-7}$
	550	3.46 × $10^{-1}$	8.96 × $10^{-7}$	2.83 × $10^{-1}$	5.65 × $10^{-7}$	3.91 × $10^{-1}$	8.37 × $10^{-7}$	2.39 × $10^{-1}$	5.05 × $10^{-7}$	4.44 × $10^{-1}$	9.68 × $10^{-7}$	4.68 × $10^{-1}$	8.28 × $10^{-7}$
	600	3.72 × $10^{-1}$	9.38 × $10^{-7}$	4.57 × $10^{-1}$	7.89 × $10^{-7}$	5.05 × $10^{-1}$	9.93 × $10^{-7}$	4.00 × $10^{-1}$	7.50 × $10^{-7}$	4.84 × $10^{-1}$	1.01 × $10^{-6}$	4.98 × $10^{-1}$	8.52 × $10^{-7}$
	650	3.71 × $10^{-1}$	9.58 × $10^{-7}$	4.36 × $10^{-1}$	8.26 × $10^{-7}$	5.63 × $10^{-1}$	1.17 × $10^{-6}$	5.20 × $10^{-1}$	9.93 × $10^{-7}$	5.37 × $10^{-1}$	1.04 × $10^{-6}$	5.22 × $10^{-1}$	8.69 × $10^{-7}$
	700	3.53 × $10^{-1}$	9.46 × $10^{-7}$	3.38 × $10^{-1}$	7.21 × $10^{-7}$	5.98 × $10^{-1}$	1.35 × $10^{-6}$	5.33 × $10^{-1}$	1.17 × $10^{-6}$	5.60 × $10^{-1}$	1.05 × $10^{-6}$	5.08 × $10^{-1}$	8.81 × $10^{-7}$
Resonance wavelengths	64	5.10 × $10^{-26}$	3.83 × $10^{-32}$	3.51 × $10^{-26}$	1.99 × $10^{-32}$	9.73 × $10^{-26}$	1.34 × $10^{-31}$	6.68 × $10^{-27}$	7.25 × $10^{-33}$	9.03 × $10^{-26}$	1.56 × $10^{-31}$	7.76 × $10^{-26}$	5.50 × $10^{-32}$
	238	6.68 × $10^{-9}$	1.62 × $10^{-14}$	3.87 × $10^{-9}$	6.46 × $10^{-15}$	7.29 × $10^{-9}$	1.43 × $10^{-14}$	1.19 × $10^{-9}$	1.63 × $10^{-15}$	9.91 × $10^{-9}$	2.25 × $10^{-14}$	8.22 × $10^{-9}$	1.49 × $10^{-14}$
	536	3.26 × $10^{-1}$	8.65 × $10^{-7}$	2.34 × $10^{-1}$	5.21 × $10^{-7}$	3.57 × $10^{-1}$	7.60 × $10^{-7}$	1.94 × $10^{-1}$	4.00 × $10^{-7}$	4.22 × $10^{-1}$	9.37 × $10^{-7}$	4.48 × $10^{-1}$	8.03 × $10^{-7}$

**Table A9:** Summary table of the results obtained for the gold nanostructures—Probe 3.

		Rectangle Thickness = 20 nm		Rectangle Thickness = 50 nm		Circle r100		Circle r200		Triangle Base = 100 nm		Triangle Base = 200 nm	
	λ **[nm]**	$|E_{\max}|$ **[V/m]**	∫Edx	$|E_{\max}|$ **[V/m]**	∫Edx	$|E_{\max}|$ **[V/m]**	∫Edx	$|E_{\max}|$ **[V/m]**	∫Edx	$|E_{\max}|$ **[V/m]**	∫Edx	$|E_{\max}|$ **[V/m]**	∫Edx
UV	250	7.50 × $10^{-61}$	1.65 × $10^{-66}$	3.11 × $10^{-61}$	6.50 × $10^{-67}$	5.28 × $10^{-61}$	1.09 × $10^{-66}$	7.23 × $10^{-62}$	1.27 × $10^{-67}$	1.06 × $10^{-60}$	2.24 × $10^{-66}$	6.64 × $10^{-61}$	1.49 × $10^{-66}$
	300	4.17 × $10^{-58}$	9.27 × $10^{-64}$	1.94 × $10^{-58}$	4.26 × $10^{-64}$	3.67 × $10^{-58}$	6.80 × $10^{-64}$	5.40 × $10^{-59}$	1.09 × $10^{-64}$	5.00 × $10^{-58}$	1.11 × $10^{-63}$	3.73 × $10^{-58}$	7.98 × $10^{-64}$
	350	3.00 × $10^{-36}$	7.87 × $10^{-42}$	1.56 × $10^{-36}$	4.19 × $10^{-42}$	2.51 × $10^{-36}$	5.34 × $10^{-42}$	4.88 × $10^{-37}$	9.41 × $10^{-43}$	3.28 × $10^{-36}$	8.84 × $10^{-42}$	2.62 × $10^{-36}$	6.78 × $10^{-42}$
Visible Light	400	3.67 × $10^{-5}$	9.87 × $10^{-11}$	2.65 × $10^{-5}$	6.04 × $10^{-11}$	4.05 × $10^{-5}$	6.60 × $10^{-11}$	8.00 × $10^{-6}$	1.69 × $10^{-11}$	4.12 × $10^{-5}$	1.10 × $10^{-10}$	3.23 × $10^{-5}$	8.72 × $10^{-11}$
	450	1.57 × $10^{-2}$	4.32 × $10^{-8}$	1.11 × $10^{-2}$	2.81 × $10^{-8}$	1.62 × $10^{-2}$	2.07 × $10^{-8}$	4.04 × $10^{-3}$	4.27 × $10^{-9}$	1.91 × $10^{-2}$	4.82 × $10^{-8}$	1.46 × $10^{-2}$	3.87 × $10^{-8}$
	500	9.67 × $10^{-2}$	2.73 × $10^{-7}$	6.49 × $10^{-2}$	1.70 × $10^{-7}$	9.56 × $10^{-2}$	1.82 × $10^{-7}$	3.41 × $10^{-2}$	5.05 × $10^{-8}$	1.18 × $10^{-1}$	3.09 × $10^{-7}$	1.18 × $10^{-1}$	2.77 × $10^{-7}$
	550	2.02 × $10^{-1}$	5.28 × $10^{-7}$	1.53 × $10^{-1}$	3.29 × $10^{-7}$	1.99 × $10^{-1}$	4.90 × $10^{-7}$	1.14 × $10^{-1}$	2.86 × $10^{-7}$	2.48 × $10^{-1}$	5.91 × $10^{-7}$	2.65 × $10^{-1}$	5.18 × $10^{-7}$
	600	2.55 × $10^{-1}$	6.69 × $10^{-7}$	2.56 × $10^{-1}$	5.55 × $10^{-7}$	2.90 × $10^{-1}$	7.28 × $10^{-7}$	2.48 × $10^{-1}$	5.20 × $10^{-7}$	3.68 × $10^{-1}$	7.46 × $10^{-7}$	3.29 × $10^{-1}$	6.47 × $10^{-7}$
	650	3.31 × $10^{-1}$	8.03 × $10^{-7}$	3.99 × $10^{-1}$	6.17 × $10^{-7}$	4.41 × $10^{-1}$	1.00 × $10^{-6}$	3.52 × $10^{-1}$	8.57 × $10^{-7}$	3.94 × $10^{-1}$	9.20 × $10^{-7}$	3.97 × $10^{-1}$	7.95 × $10^{-7}$
	700	3.27 × $10^{-1}$	8.44 × $10^{-7}$	2.91 × $10^{-1}$	6.38 × $10^{-7}$	6.45 × $10^{-1}$	1.17 × $10^{-6}$	5.72 × $10^{-1}$	1.01 × $10^{-6}$	4.81 × $10^{-1}$	1.00 × $10^{-6}$	4.25 × $10^{-1}$	8.69 × $10^{-7}$
Resonance wavelengths	64	7.44 × $10^{-151}$	1.24 × $10^{-157}$	5.64 × $10^{-153}$	7.49 × $10^{-160}$	2.87 × $10^{-150}$	6.99 × $10^{-157}$	3.63 × $10^{-152}$	1.17 × $10^{-158}$	2.12 × $10^{-151}$	5.64 × $10^{-158}$	2.85 × $10^{-151}$	3.52 × $10^{-158}$
	238	4.81 × $10^{-59}$	1.05 × $10^{-64}$	2.00 × $10^{-59}$	4.17 × $10^{-65}$	3.76 × $10^{-59}$	7.81 × $10^{-65}$	4.36 × $10^{-60}$	7.70 × $10^{-66}$	6.67 × $10^{-59}$	1.42 × $10^{-64}$	4.43 × $10^{-59}$	1.00 × $10^{-64}$
	536	1.65 × $10^{-1}$	4.34 × $10^{-7}$	1.13 × $10^{-1}$	2.61 × $10^{-7}$	1.39 × $10^{-1}$	3.76 × $10^{-7}$	7.38 × $10^{-2}$	1.92 × $10^{-7}$	1.93 × $10^{-1}$	4.86 × $10^{-7}$	2.14 × $10^{-1}$	4.30 × $10^{-7}$

**Table A10:** Summary table of the results obtained for the copper nanostructures—Probe 1.

		Rectangle Thickness = 20 nm		Rectangle Thickness = 50 nm		Circle r100		Circle r200		Triangle Base = 100 nm		Triangle Base = 200 nm	
	λ **[nm]**	$|E_{\max}|$ **[V/m]**	∫Edx	$|E_{\max}|$ **[V/m]**	∫Edx	$|E_{\max}|$ **[V/m]**	∫Edx	$|E_{\max}|$ **[V/m]**	∫Edx	$|E_{\max}|$ **[V/m]**	∫Edx	$|E_{\max}|$ **[V/m]**	∫Edx
UV	250	2.46 × $10^{-2}$	4.03 × $10^{-8}$	1.41 × $10^{-2}$	1.49 × $10^{-8}$	2.08 × $10^{-2}$	4.13 × $10^{-8}$	6.33 × $10^{-3}$	1.06 × $10^{-8}$	3.65 × $10^{-2}$	6.02 × $10^{-8}$	2.11 × $10^{-2}$	3.41 × $10^{-8}$
	300	1.80 × $10^{-2}$	3.60 × $10^{-8}$	1.34 × $10^{-2}$	1.73 × $10^{-8}$	1.91 × $10^{-2}$	3.09 × $10^{-8}$	3.33 × $10^{-3}$	7.05 × $10^{-9}$	1.93 × $10^{-2}$	4.27 × $10^{-8}$	1.55 × $10^{-2}$	3.07 × $10^{-8}$
	350	5.21 × $10^{-2}$	1.08 × $10^{-7}$	4.28 × $10^{-2}$	5.45 × $10^{-8}$	5.13 × $10^{-2}$	1.07 × $10^{-7}$	1.11 × $10^{-2}$	2.70 × $10^{-8}$	5.47 × $10^{-2}$	1.26 × $10^{-7}$	4.44 × $10^{-2}$	9.10 × $10^{-8}$
Visible Light	400	2.58 × $10^{-1}$	5.40 × $10^{-7}$	2.45 × $10^{-1}$	2.98 × $10^{-7}$	2.00 × $10^{-1}$	5.20 × $10^{-7}$	6.40 × $10^{-2}$	1.79 × $10^{-7}$	2.66 × $10^{-1}$	6.16 × $10^{-7}$	2.03 × $10^{-1}$	4.45 × $10^{-7}$
	450	3.07 × $10^{-1}$	7.34 × $10^{-7}$	2.66 × $10^{-1}$	4.26 × $10^{-7}$	2.03 × $10^{-1}$	5.47 × $10^{-7}$	6.69 × $10^{-2}$	1.85 × $10^{-7}$	3.63 × $10^{-1}$	8.60 × $10^{-7}$	2.67 × $10^{-1}$	6.33 × $10^{-7}$
	500	3.60 × $10^{-1}$	8.58 × $10^{-7}$	2.37 × $10^{-1}$	4.76 × $10^{-7}$	2.95 × $10^{-1}$	7.95 × $10^{-7}$	1.39 × $10^{-1}$	3.73 × $10^{-7}$	4.13 × $10^{-1}$	9.78 × $10^{-7}$	4.85 × $10^{-1}$	8.32 × $10^{-7}$
	550	**3.97 × $10^{-1}$**	9.16 × $10^{-7}$	4.63 × $10^{-1}$	6.45 × $10^{-7}$	4.04 × $10^{-1}$	9.94 × $10^{-7}$	2.64 × $10^{-1}$	6.81 × $10^{-7}$	4.32 × $10^{-1}$	1.03 × $10^{-6}$	4.93 × $10^{-1}$	8.65 × $10^{-7}$
	600	3.96 × $10^{-1}$	**9.33 × $10^{-7}$**	**6.09 × $10^{-1}$**	**7.65 × $10^{-7}$**	4.76 × $10^{-1}$	1.16 × $10^{-6}$	3.97 × $10^{-1}$	9.42 × $10^{-7}$	4.46 × $10^{-1}$	1.06 × $10^{-6}$	4.98 × $10^{-1}$	8.72 × $10^{-7}$
	650	3.87 × $10^{-1}$	9.24 × $10^{-7}$	5.72 × $10^{-1}$	6.81 × $10^{-7}$	5.48 × $10^{-1}$	1.32 × $10^{-6}$	4.90 × $10^{-1}$	1.18 × $10^{-6}$	4.54 × $10^{-1}$	1.07 × $10^{-6}$	**5.12 × $10^{-1}$**	8.71 × $10^{-7}$
	700	3.61 × $10^{-1}$	8.99 × $10^{-7}$	4.62 × $10^{-1}$	6.05 × $10^{-7}$	**6.49 × $10^{-1}$**	**1.45 × $10^{-6}$**	**5.45 × $10^{-1}$**	**1.33 × $10^{-6}$**	**4.66 × $10^{-1}$**	**1.09 × $10^{-6}$**	5.01 × $10^{-1}$	**8.72 × $10^{-7}$**
Resonance wavelengths	100	1.15 × $10^{-3}$	9.58 × $10^{-10}$	1.01 × $10^{-3}$	5.07 × $10^{-10}$	1.85 × $10^{-3}$	2.75 × $10^{-9}$	2.24 × $10^{-4}$	2.86 × $10^{-10}$	2.23 × $10^{-3}$	3.08 × $10^{-9}$	1.65 × $10^{-3}$	1.27 × $10^{-9}$
	158	9.41 × $10^{-3}$	1.33 × $10^{-8}$	7.84 × $10^{-3}$	5.07 × $10^{-9}$	1.44 × $10^{-2}$	2.52 × $10^{-8}$	1.83 × $10^{-3}$	3.28 × $10^{-9}$	2.11 × $10^{-2}$	2.77 × $10^{-8}$	1.40 × $10^{-2}$	1.42 × $10^{-8}$
	360	5.43 × $10^{-2}$	1.11 × $10^{-7}$	4.55 × $10^{-2}$	5.64 × $10^{-8}$	4.97 × $10^{-2}$	1.10 × $10^{-7}$	1.26 × $10^{-2}$	3.21 × $10^{-8}$	5.53 × $10^{-2}$	1.31 × $10^{-7}$	5.11 × $10^{-2}$	9.70 × $10^{-8}$
	526	3.82 × $10^{-1}$	8.93 × $10^{-7}$	3.31 × $10^{-1}$	5.49 × $10^{-7}$	3.50 × $10^{-1}$	8.99 × $10^{-7}$	2.00 × $10^{-1}$	5.34 × $10^{-7}$	4.27 × $10^{-1}$	1.01 × $10^{-6}$	4.81 × $10^{-1}$	8.50 × $10^{-7}$

**Table A11:** Summary table of the results obtained for the copper nanostructures—Probe 2.

		Retangle Thickness = 20 nm		Retangle Thickness = 50 nm		Circle r100		Circle r200		Triangle Base = 100 nm		Triangle Base = 200 nm	
	λ **[nm]**	$|E_{\max}|$ **[V/m]**	∫Edx	$|E_{\max}|$ **[V/m]**	∫Edx	$|E_{\max}|$ **[V/m]**	∫Edx	$|E_{\max}|$ **[V/m]**	∫Edx	$|E_{\max}|$ **[V/m]**	∫Edx	$|E_{\max}|$ **[V/m]**	∫Edx
UV	250	4.02 × $10^{-9}$	8.52 × $10^{-15}$	2.05 × $10^{-9}$	3.15 × $10^{-15}$	4.20 × $10^{-9}$	7.76 × $10^{-15}$	1.10 × $10^{-9}$	1.65 × $10^{-15}$	5.97 × $10^{-9}$	1.26 × $10^{-14}$	4.08 × $10^{-9}$	7.31 × $10^{-15}$
	300	6.41 × $10^{-9}$	1.52 × $10^{-14}$	4.44 × $10^{-9}$	7.27 × $10^{-15}$	7.55 × $10^{-9}$	1.25 × $10^{-14}$	1.20 × $10^{-9}$	2.57 × $10^{-15}$	7.38 × $10^{-9}$	1.80 × $10^{-14}$	6.37 × $10^{-9}$	1.30 × $10^{-14}$
	350	5.11 × $10^{-6}$	1.25 × $10^{-11}$	3.86 × $10^{-6}$	6.26 × $10^{-12}$	5.44 × $10^{-6}$	1.14 × $10^{-11}$	1.08 × $10^{-6}$	2.74 × $10^{-12}$	5.82 × $10^{-6}$	1.45 × $10^{-11}$	5.08 × $10^{-6}$	1.05 × $10^{-11}$
Visible Light	400	7.73 × $10^{-2}$	1.96 × $10^{-7}$	6.45 × $10^{-2}$	1.13 × $10^{-7}$	8.82 × $10^{-2}$	1.81 × $10^{-7}$	2.97 × $10^{-2}$	6.15 × $10^{-8}$	9.07 × $10^{-2}$	2.24 × $10^{-7}$	7.56 × $10^{-2}$	1.61 × $10^{-7}$
	450	1.92 × $10^{-1}$	5.20 × $10^{-7}$	1.36 × $10^{-1}$	3.09 × $10^{-7}$	1.74 × $10^{-1}$	3.74 × $10^{-7}$	5.92 × $10^{-2}$	1.26 × $10^{-7}$	2.80 × $10^{-1}$	5.99 × $10^{-7}$	2.04 × $10^{-1}$	4.41 × $10^{-7}$
	500	2.80 × $10^{-1}$	7.40 × $10^{-7}$	1.80 × $10^{-1}$	4.08 × $10^{-7}$	3.23 × $10^{-1}$	6.60 × $10^{-7}$	1.56 × $10^{-1}$	3.05 × $10^{-7}$	3.96 × $10^{-1}$	8.38 × $10^{-7}$	4.33 × $10^{-1}$	6.98 × $10^{-7}$
	550	3.40 × $10^{-1}$	8.49 × $10^{-7}$	3.43 × $10^{-1}$	6.14 × $10^{-7}$	4.13 × $10^{-1}$	8.93 × $10^{-7}$	2.88 × $10^{-1}$	6.07 × $10^{-7}$	4.63 × $10^{-1}$	9.45 × $10^{-7}$	4.77 × $10^{-1}$	7.81 × $10^{-7}$
	600	3.61 × $10^{-1}$	8.85 × $10^{-7}$	4.59 × $10^{-1}$	7.90 × $10^{-7}$	5.19 × $10^{-1}$	1.03 × $10^{-6}$	4.34 × $10^{-1}$	8.23 × $10^{-7}$	5.00 × $10^{-1}$	9.87 × $10^{-7}$	5.03 × $10^{-1}$	8.06 × $10^{-7}$
	650	3.52 × $10^{-1}$	8.97 × $10^{-7}$	3.90 × $10^{-1}$	7.44 × $10^{-7}$	5.81 × $10^{-1}$	1.22 × $10^{-6}$	5.33 × $10^{-1}$	1.07 × $10^{-6}$	5.54 × $10^{-1}$	1.01 × $10^{-6}$	5.23 × $10^{-1}$	8.27 × $10^{-7}$
	700	3.29 × $10^{-1}$	8.73 × $10^{-7}$	2.79 × $10^{-1}$	6.00 × $10^{-7}$	6.34 × $10^{-1}$	1.40 × $10^{-6}$	5.72 × $10^{-1}$	1.20 × $10^{-6}$	5.73 × $10^{-1}$	1.03 × $10^{-6}$	5.03 × $10^{-1}$	8.44 × $10^{-7}$
Resonance wavelengths	100	3.78 × $10^{-17}$	4.22 × $10^{-23}$	3.21 × $10^{-17}$	2.20 × $10^{-23}$	7.90 × $10^{-17}$	1.13 × $10^{-22}$	7.24 × $10^{-18}$	9.18 × $10^{-24}$	8.17 × $10^{-17}$	1.36 × $10^{-22}$	6.44 × $10^{-17}$	5.70 × $10^{-23}$
	158	2.54 × $10^{-11}$	4.72 × $10^{-17}$	1.82 × $10^{-11}$	1.75 × $10^{-17}$	4.18 × $10^{-11}$	7.89 × $10^{-17}$	5.69 × $10^{-12}$	8.73 × $10^{-18}$	5.85 × $10^{-11}$	9.75 × $10^{-17}$	4.17 × $10^{-11}$	5.08 × $10^{-17}$
	360	9.27 × $10^{-6}$	2.22 × $10^{-11}$	7.16 × $10^{-6}$	1.12 × $10^{-11}$	9.18 × $10^{-6}$	2.05 × $10^{-11}$	2.14 × $10^{-6}$	5.70 × $10^{-12}$	1.08 × $10^{-5}$	2.61 × $10^{-11}$	1.02 × $10^{-5}$	1.93 × $10^{-11}$
	526	3.10 × $10^{-1}$	8.00 × $10^{-7}$	2.42 × $10^{-1}$	4.85 × $10^{-7}$	3.69 × $10^{-1}$	7.80 × $10^{-7}$	2.26 × $10^{-1}$	4.54 × $10^{-7}$	4.25 × $10^{-1}$	8.95 × $10^{-7}$	4.47 × $10^{-1}$	7.43 × $10^{-7}$

**Table A12:** Summary table of the results obtained for the copper nanostructures—Probe 3.

		Retangle Thickness = 20 nm		Retangle Thickness = 50 nm		Circle r100		Circle r200		Triangle Base = 100 nm		Triangle Base = 200 nm	
	λ **[nm]**	$|E_{\max}|$ **[V/m]**	∫Edx	$|E_{\max}|$ **[V/m]**	∫Edx	$|E_{\max}|$ **[V/m]**	∫Edx	$|E_{\max}|$ **[V/m]**	∫Edx	$|E_{\max}|$ **[V/m]**	∫Edx	$|E_{\max}|$ **[V/m]**	∫Edx
UV	250	6.93 × $10^{-61}$	1.53 × $10^{-66}$	2.66 × $10^{-61}$	5.57 × $10^{-67}$	5.97 × $10^{-61}$	1.23 × $10^{-66}$	1.09 × $10^{-61}$	1.91 × $10^{-67}$	1.04 × $10^{-60}$	2.21 × $10^{-66}$	5.97 × $10^{-61}$	1.35 × $10^{-66}$
	300	4.24 × $10^{-58}$	9.41 × $10^{-64}$	1.98 × $10^{-58}$	4.32 × $10^{-64}$	3.64 × $10^{-58}$	6.73 × $10^{-64}$	4.99 × $10^{-59}$	9.97 × $10^{-65}$	5.02 × $10^{-58}$	1.11 × $10^{-63}$	3.80 × $10^{-58}$	8.11 × $10^{-64}$
	350	2.87 × $10^{-36}$	7.40 × $10^{-42}$	1.45 × $10^{-36}$	3.55 × $10^{-42}$	2.47 × $10^{-36}$	5.18 × $10^{-42}$	5.45 × $10^{-37}$	1.03 × $10^{-42}$	3.32 × $10^{-36}$	8.60 × $10^{-42}$	2.52 × $10^{-36}$	6.22 × $10^{-42}$
Visible Light	400	3.45 × $10^{-5}$	9.11 × $10^{-11}$	2.41 × $10^{-5}$	5.19 × $10^{-11}$	4.29 × $10^{-5}$	6.90 × $10^{-11}$	1.11 × $10^{-5}$	2.35 × $10^{-11}$	4.12 × $10^{-5}$	1.05 × $10^{-10}$	2.86 × $10^{-5}$	7.72 × $10^{-11}$
	450	1.49 × $10^{-2}$	3.98 × $10^{-8}$	9.81 × $10^{-3}$	2.32 × $10^{-8}$	1.79 × $10^{-2}$	2.22 × $10^{-8}$	6.20 × $10^{-3}$	6.35 × $10^{-9}$	1.97 × $10^{-2}$	4.72 × $10^{-8}$	1.42 × $10^{-2}$	3.41 × $10^{-8}$
	500	9.04 × $10^{-2}$	2.57 × $10^{-7}$	5.72 × $10^{-2}$	1.43 × $10^{-7}$	1.04 × $10^{-1}$	2.13 × $10^{-7}$	5.75 × $10^{-2}$	9.01 × $10^{-8}$	1.21 × $10^{-1}$	3.05 × $10^{-7}$	1.27 × $10^{-1}$	2.71 × $10^{-7}$
	550	1.98 × $10^{-1}$	4.99 × $10^{-7}$	1.98 × $10^{-1}$	3.37 × $10^{-7}$	2.12 × $10^{-1}$	5.23 × $10^{-7}$	1.39 × $10^{-1}$	3.44 × $10^{-7}$	2.56 × $10^{-1}$	5.79 × $10^{-7}$	2.72 × $10^{-1}$	4.93 × $10^{-7}$
	600	2.45 × $10^{-1}$	6.29 × $10^{-7}$	2.58 × $10^{-1}$	5.50 × $10^{-7}$	2.99 × $10^{-1}$	7.55 × $10^{-7}$	2.70 × $10^{-1}$	5.72 × $10^{-7}$	3.77 × $10^{-1}$	7.30 × $10^{-7}$	3.22 × $10^{-1}$	6.18 × $10^{-7}$
	650	3.15 × $10^{-1}$	7.50 × $10^{-7}$	3.60 × $10^{-1}$	5.62 × $10^{-7}$	4.69 × $10^{-1}$	1.04 × $10^{-6}$	3.76 × $10^{-1}$	9.15 × $10^{-7}$	3.95 × $10^{-1}$	9.05 × $10^{-7}$	3.95 × $10^{-1}$	7.70 × $10^{-7}$
	700	3.09 × $10^{-1}$	7.77 × $10^{-7}$	2.45 × $10^{-1}$	5.30 × $10^{-7}$	6.67 × $10^{-1}$	1.20 × $10^{-6}$	5.83 × $10^{-1}$	1.04 × $10^{-6}$	4.86 × $10^{-1}$	9.89 × $10^{-7}$	4.17 × $10^{-1}$	8.45 × $10^{-7}$
Resonance wavelengths	100	1.95 × $10^{-117}$	3.78 × $10^{-124}$	3.07 × $10^{-118}$	1.21 × $10^{-124}$	5.04 × $10^{-117}$	1.22 × $10^{-123}$	4.30 × $10^{-118}$	1.08 × $10^{-124}$	2.48 × $10^{-117}$	1.23 × $10^{-123}$	3.10 × $10^{-117}$	8.48 × $10^{-124}$
	158	6.49 × $10^{-77}$	1.22 × $10^{-82}$	2.70 × $10^{-77}$	4.46 × $10^{-83}$	1.05 × $10^{-76}$	1.85 × $10^{-82}$	1.30 × $10^{-77}$	1.95 × $10^{-83}$	1.40 × $10^{-76}$	2.52 × $10^{-82}$	8.10 × $10^{-77}$	1.39 × $10^{-82}$
	360	4.79 × $10^{-34}$	1.19 × $10^{-39}$	2.47 × $10^{-34}$	5.62 × $10^{-40}$	4.29 × $10^{-34}$	8.49 × $10^{-40}$	1.12 × $10^{-34}$	1.99 × $10^{-40}$	5.77 × $10^{-34}$	1.40 × $10^{-39}$	4.62 × $10^{-34}$	1.03 × $10^{-39}$
	526	1.42 × $10^{-1}$	3.66 × $10^{-7}$	1.08 × $10^{-1}$	2.18 × $10^{-7}$	1.32 × $10^{-1}$	3.51 × $10^{-7}$	8.14 × $10^{-2}$	1.96 × $10^{-7}$	1.73 × $10^{-1}$	4.27 × $10^{-7}$	1.94 × $10^{-1}$	3.70 × $10^{-7}$
